# Catalytic Activity of Cellulose‐Supported Platinum and Palladium Nanoparticles for Allylbenzene Hydrogenation

**DOI:** 10.1002/chem.202402952

**Published:** 2024-12-12

**Authors:** Tabea Angela Thiel, Riny Yolandha Parapat, Michael Schroeter, Michael Schwarze

**Affiliations:** ^1^ Department of Chemistry Technische Universität Berlin TC8, Straße des 17. Juni 124 10623 Berlin Germany; ^2^ Leibniz Institute for Catalysis Albert-Einstein-Straße 29a 18059 Rostock Germany; ^3^ Department of Chemical Engineering Institut Teknologi Nasional Bandung (ITENAS) PHH Mustopha 23 40124 Bandung Indonesia; ^4^ Institute of Functional Materials for Sustainability Helmholtz-Zentrum Hereon, Kantstrasse 55 14513 Teltow Germany

**Keywords:** Allylbenzene, Cellulose, Hydrogenation, Metal nanoparticles, Supported catalyst

## Abstract

Platinum and palladium nanoparticles were successfully deposited on tunicate cellulose via the photodeposition or microemulsion deposition method. Evenly distributed, small and narrow‐sized particles in the range of 2–3 nm were obtained for microemulsion‐prepared cellulose catalysts. The photodeposition method led to larger particle sizes, broader size distribution, and occasional agglomerations. The catalysts were tested in the allylbenzene hydrogenation reaction at room temperature and the results were compared to commercially available catalysts. Because of smaller particles, both microemulsion‐prepared catalysts and photodeposited ones show better activity than commercial catalysts. Even platinum and palladium nanoparticles were active for the hydrogenation, only cellulose‐supported platinum nanoparticles showed good stability. For palladium nanoparticles, stronger leaching from the surface of cellulose was observed. Cellulose‐supported catalysts were recycled, and reusability is comparable to commercial catalysts. Therefore, cellulose could be used as an alternative catalyst support.

## Graphical abstract

Platinum or palladium nanoparticles were immobilized on cellulose using a colloidal deposition method and their catalytic performance for the hydrogenation of allylbenzene was investigated. Compared to the commercial catalyst, the activities are higher, while the stability of the catalysts immobilized on cellulose was better for the platinum nanoparticles.

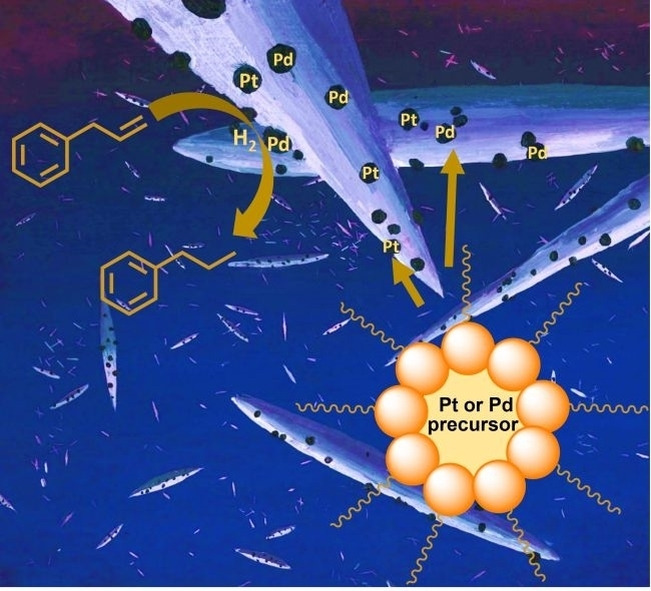

## Introduction

Green chemistry has become increasingly important in overcoming environmental problems and climate change in the past 20 years. It is defined by twelve principles, including catalysis, energy efficiency, atom economy, renewable feedstocks, less hazardous synthesis pathways and chemicals, waste prevention, atom economy, the design of benign chemicals, green solvents, and auxiliaries, reducing derivatives, degradability, real‐time analysis for pollution prevention and overall accident prevention.^[1]^ Catalysis plays a key role in Green chemistry, with the general aim to lower the required energy input and to increase the efficiency of a chemical reaction. In this direction, the immobilization of active compounds like metal complexes in homogeneous catalysis or metal nanoparticles in heterogeneous catalysis is a major step to facilitate catalyst separation and recycling. Focusing on supported metal nanoparticles, metal oxides, e. g. alumina Al_2_O_3_ and silica SiO_2_, are preferred as support materials due to their high chemical and thermal stability.[^2^, ^3^] As many reactions, such as cross‐coupling reactions e. g. Suzuki,[^4^, ^5^] Heck^[5]^ and Ullmann,^[6]^ and hydrogenation of phenol^[7]^ or allylbenzene,^[8]^ take place at lower temperatures, a high thermal stability of the support material is not always mandatory. This enables the use of sustainable biomaterial supports like chitosan, starch, lignin, and cellulose as attractive alternatives to conventional metal oxides.[^3^, ^9^, ^10^, ^11^] Cellulose is the most abundant natural polymer and has been investigated as a support material for metal nanoparticles since the last decade. The use of cellulose as a catalyst support in the deposition of various metal (silver (Ag), gold (Au), platinum (Pt), palladium (Pd), copper (Cu) and metal oxide (e. g. CuO) nanoparticles is widely published.[^5^, ^12^] A large scope of different metals deposited as nanoparticles onto cellulose, such as Ag, Ni, Au, Cu, CuO, Au−Pd, Pt, and Pd, were investigated for para‐nitrophenol reduction with sodium borohydride (NaBH_4_) as the reductant.[^12^, ^13^] Hydrogenations with cellulose‐supported metal catalysts were investigated by the Moores group.^[11]^ Cellulose‐supported Pd catalysts were applied in Suzuki,[^14^, ^15^] Heck,[^7^, ^16^, ^17^] and Ullmann coupling.^[6]^ Furthermore, those Pd catalysts were applied for hydrogenations of phenol to cyclohexanone^[7]^ and benzaldehyde derivatives to their respective secondary alcohols.^[18]^ Cellulose‐supported Ag catalysts were used for the reduction of acetophenone derivatives to their respective alcohols,^[19]^ phenylacetylene to ethylbenzene,^[19]^ and benzene to cyclohexene.^[20]^ As cellulose shows thermal degradation at higher temperatures, immobilization methods that include a calcination step, e. g. classical impregnation, are unsuitable. However, different methods have been reported to deposit metal nanoparticles from metal salt precursors onto cellulose. The precursor can be reduced with external reducing agents such as sodium borohydride (NaBH_4_), hydrogen (H_2_), ascorbic acid (AA), and thiourea (TU).^[13]^ Cellulose can be modified with reductive groups, working well for Au and Ag nanoparticle deposition. The precursor HAuCl_4_ was reported to be reduced by functionalized cellulose surfaces with thiol,^[21]^ carboxylate,^[22]^ and amine^[23]^ groups. AgNO_3_ was reported to be reduced by dopamine‐coated cellulose NP^[24]^ and aldehyde groups.^[25]^ Even without surface modification, the naturally present hydroxyl groups of cellulose can reduce metal salts suitable for a broader range of metal nanoparticles for which supercritical carbon dioxide (scCO_2_) can be used as a solvent with the advantage of easy product separation by releasing the CO_2_ pressure.^[13]^

We recently reported the photodeposition of Pt particles onto cellulose for the hydrogenation of para‐nitro phenol.^[26]^ Photodeposition is a popular deposition method applied in photocatalysis to immobilize metal nanoparticles onto semiconductors. It is mostly considered that the reduction is initiated by a surface‐trapped electron originating from light irradiation of the semiconductor. However, hexachloroplatinic acid (H_2_PtCl_6_), chloroauric acid (HAuCl_4_), silver perchlorate (AgClO_4_,), and copper acetylacetonate (Cu(acac)_2_) can be directly photochemically reduced with alcohols like ethanol and methanol to obtain the respective metal nanoparticles.^[27]^ Since alcohols can be used simultaneously as a reducing agent and solvent, the photodeposition is a very simple and green method. But, one disadvantage which was observed in our previous research, was the challenging control of the size and size distribution of the metal nanoparticles synthesized via Photodeposition.^[26]^ Microemulsions can be used to overcome the limitations of photodepostion, but they only act as a solvent for the reduction process and an external reductant is required. Microemulsions are mixtures of oil, water, and surfactant, which appear as a single, clear, transparent phase. The metal salt precursor and the reducing agent are dissolved in two separate microemulsions, which are mixed to initiate the reduction process. Commonly are water‐in‐oil microemulsions, with inverse micelles as microreactors, containing a limited amount of metal salt and reductant in a water droplet. Two inverse micelles collide and merge, leading to oversaturation, nucleation, and nanoparticle formation. The nanoparticles precipitate when they get larger than the micelle. Metal nanoparticles obtained by the microemulsion method have been reported to have a more controllable, narrow size distribution compared to the classic impregnation and precipitation methods.[^2^, ^3^, ^28^] In the preparation of supported nanoparticles using microemulsion, the initial step involves synthesizing the nanoparticles inside the micelles. Following synthesis, these nanoparticles are deposited onto a separate support material through various techniques such as impregnation, pH control, and thermal destabilization. Parapat et al. reported the deposition of various metals onto different types of alumina and silica via thermal destabilization of microemulsion.^[29]^ Their study revealed that the amount of deposited nanoparticles and their dispersion on the support are significantly influenced by differences in zeta potential values between the nanoparticles and the support material. A greater difference in zeta potential values generally leads to improved deposition and stability. Additionally, they emphasized the crucial roles played by the characteristics of the nanoparticles including size and shape and the properties of the support material such as pore size and surface area in determining the effectiveness of the deposition process.[^30^, ^31^] In this work, Pd and Pt nanoparticles were prepared and immobilized onto cellulose as sustainable biobased support material via two methods: photodeposition and microemulsion deposition. The Pd and Pt nanoparticles were investigated regarding their morphology, size distribution, crystallinity, dispersion, and catalyst loading. The catalysts were applied in the hydrogenation of allylbenzene to propylbenzene as a model reaction for heterogeneously catalyzed C−C double‐bond hydrogenation. Although the para‐nitrophenol (PNP) reduction with NaBH_4_ is a popular model reaction, due to its simple and less time‐consuming implementation by monitoring the reaction with UV‐vis, we decided to use the ALB hydrogenation as model reaction. The reaction progress can be directly monitored by the hydrogen consumption because it needs only one equivalent of hydrogen to reduce one equivalent of ALB to propylbenzene (PB). In contrast, one equivalent of PNP needs three equivalents of hydrogen for its reduction to para‐aminophenol. Additionally, we found that the PNP reduction is quite sensitive due to its small reaction volume (up to 3 mL) and is aside from UV‐Vis hard to monitor via GC or HPLC due to a larger number of intermediates. Besides catalyst characterization and activity, a focus was on catalyst leaching and recycling, and as well as the question of the applicability of a cellulose‐supported catalyst. Commercially available alumina‐supported catalysts were used as references to conclude whether and in which manner the catalyst characteristics and reaction performance differ from the commercial catalysts.

## Results and Discussion

### Morphology, Size Distribution, and Catalyst Loading

**Figure 1:**
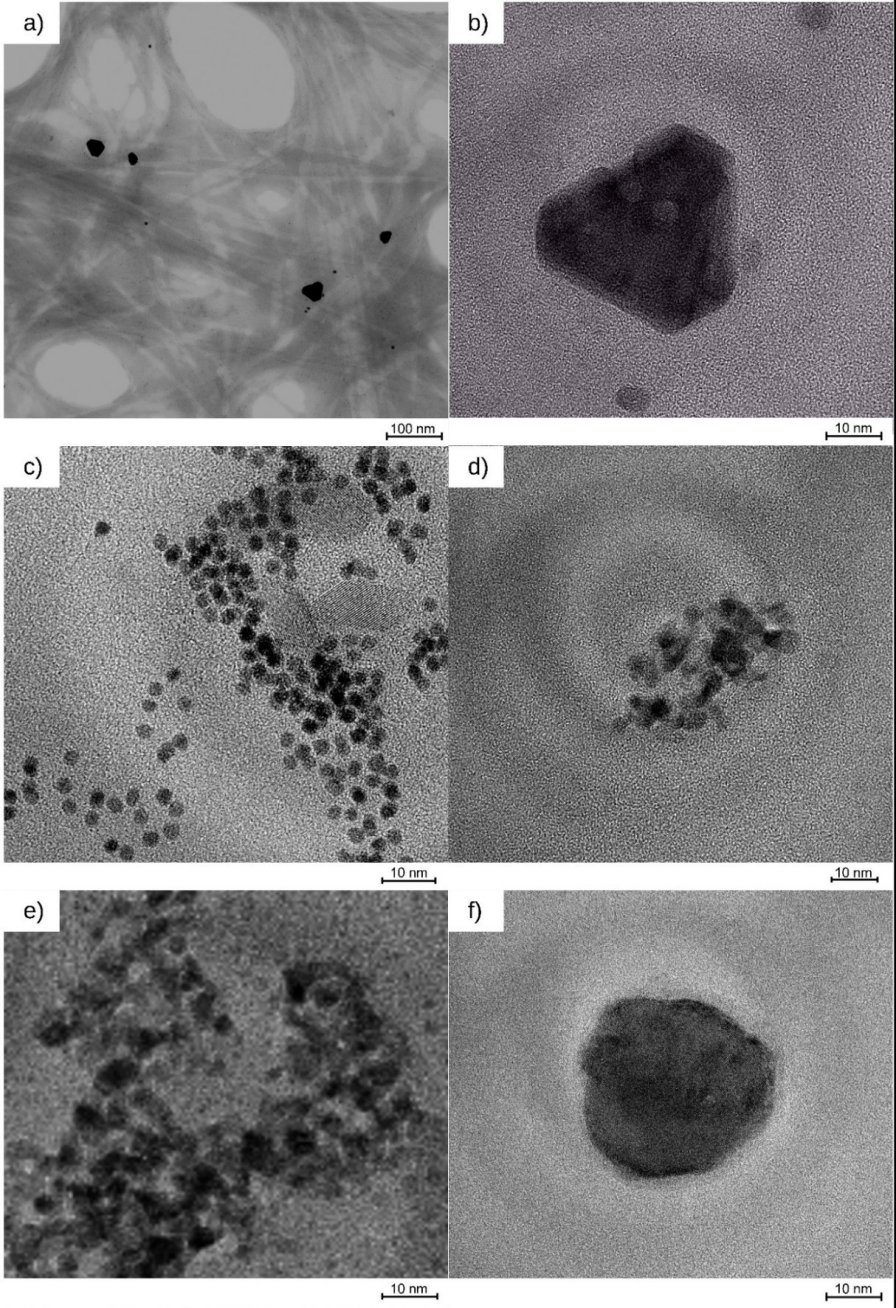
HR‐TEM images of PDPd (a and b), MEPt (c), MEPd1 (d), MEPd2 (e), and MEPd2 after a hydrogenation experiment (f).

**Figure 2:**
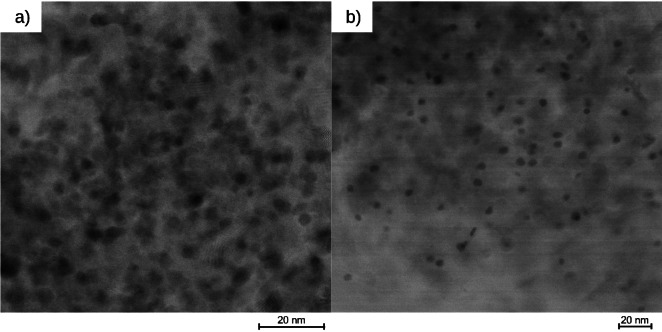
STEM‐BF images of commercial Pd@Al_2_O_3_ (a) and Pt@Al_2_O_3_ (b) catalysts.

Catalysts were prepared via photodeposition (PD) and microemulsion deposition (ME). The prepared catalyst and the commercial catalysts Pd@Al_2_O_3_ and Pt@Al_2_O_3_ were characterized by their loadings, NP sizes and size distributions, and crystallinity (Table 1). The size and morphology of Pd and Pt NPs for synthesized and commercial catalysts were investigated using (Figures 1 and 2). The loadings of the synthesized Pd@ModCe and Pt@ModCe catalysts were determined by ICP‐OES. For a better overview, the characteristic values of the prepared catalysts are summarized in Table 2. The Pt catalyst PDPt prepared by photodeposition has already been tested for the reduction of para nitro phenol (PNP).^[26]^ Nevertheless, to enable a clear overview of the following evaluations, the display of its characterization is necessary. Analog to PDPt, Pd NPs were photodeposited onto cellulose, designated as PDPd. The photodeposition of Pd NPs on cellulose has not been reported before. The photodeposition was attempted by using PdCl_2_ as a precursor in an aqueous methanol suspension of ModCe but has not been proven successful. The color of the suspension remained yellow instead of turning grey like in all other depositions, even after the irradiation of two hours, with the full spectrum of the lamp. In our previous work, we demonstrated the independence of the photodeposition of H_2_PtCl_6_ onto cellulose from the semiconductor in the presence of alcohol.^[26]^ The semi‐conductor‐free photodeposition of NPs from metal‐salt precursors such as chloroauric acid, silver perchlorate, and the chelate complex copper acetylacetonate is published Majima et al.^[27]^ Probably due to the structural relationship of K_2_PdCl_6_ to H_2_PtCl_6_, the photoreduction of K_2_PdCl_6_ with methanol is also possible.

**Table 1:** Overview of investigated catalysts, including preparing methods, average particle sizes d_NP_, the variance of the particle size σ, theoretical loadings, and loadings determined by ICP‐OES.

Catalyst	Preparation method	Metal/Precursor	d_NP_ (nm)	σ (nm)	Theoretical loading (wt %)	Loading (wt %)
PDPt^[26]^	PD	Pt/H_2_PtCl_6_	4.6	0.9	6.4	2.7
PDPd	PD	Pd/K_2_PdCl_6_	7.4	1.6	0.3–1.1	0.7
MEPt	ME	Pt/H_2_PtCl_6_	3.2	0.4	1.0	0.4
MEPd1	ME	Pd/K_2_PdCl_6_	2.1	0.4	0.3–1.0	0.4
MEPd2	ME	Pd/PdCl_2_	2.1	0.6	1.0	0.7
Pt@Al_2_O_3_	–	Pt/–	5.8	1.3	1.0^[a]^	–
Pd@Al_2_O_3_	–	Pd/–	5.0	0.6	1.0^[a]^	–

[a] Catalyst loading according to the product sheet. PD=photodeposition, ME=microemulsion deposition.

Loading yields of 70–80 % for photodeposition for Pd and Pt NPs and 40 % for microemulsion deposition for Pt (H_2_PtCl_6_) and Pd (K_2_PdCl_6_) NPs were achieved. Tasbihi et al.^[32]^ reported similar loading yields of Pt NPs on TiO_2_ using the photodeposition method. Parapat et al.^[31]^ reported similar loading yields of Pt and Pd NPs on Al_2_O_3_ using a similar procedure of the microemulsion method. A microemulsion deposition variant with a long reaction time of twelve hours overnight at 25 °C with PdCl_2_ leads to a loading of 0.7 wt % and a deposition yield of 70 %. Therefore, the microemulsion deposition method does not generally lead to small loading yields of ~0.4 wt %. However, the deposition method does influence the loading yield. When the deposition procedure differs distinctly in temperature and time using the same method, it can influence the deposition yield. The purity of the precursor K_2_PdCl_6_ was declared to be at least 26.3 %, but the actual loadings of the catalysts PDPd and MEPd1 are higher than the theoretical minimum loading. Using K_2_PdCl_6_ as the precursor, the loadings photo deposited Pd NPs with 0.7 wt % is higher than all the microemulsion deposited Pd NPs with 0.4 wt % on ModCe.

The TEM images in Figure 1b–f have the same scale bar for better comparison. Figure S1 in #ESI also shows larger sections of selected samples. Since conventional TEM is a transmission method, the particles can only be observed in projection which allows to identify two‐dimensional shapes, but an actual three‐dimensional crystal shape can only be assumed. For the photodeposited PDPd, larger nanocrystals (14–20 nm) can be found aside from the small and round NPs, as exemplified in Figure 1a and b. The actual crystal shape could consist of a bipyramidal habitus combined with a trigonal prism or a trigonal prism as a habitus combined with a second trigonal prism. These morphologies might be a results of the large reaction time of eight hours, enabling the crystal growth by aggregation or Ostwald ripening. The particles of MEPt have a size of 10 nm and have distinct circular shapes (Figure 1c), indicating a spherical shape. Slightly more irregular round shapes of Pt NPs were found for the commercial catalyst (Figure 2b). Agglomerations of Pd Nanoparticles were found for microemulsion‐prepared MEPd1 and MEPd2 (Figure 1d and e) as well as in the commercial catalyst (Figure 2a). The NPs after the hydrogenation experiment with MEPd2 (Figure 1f) have an irregular shape and a similar particle size to PDPd, which is very probably caused by the agglomeration of smaller particles.

**Figure 3:**
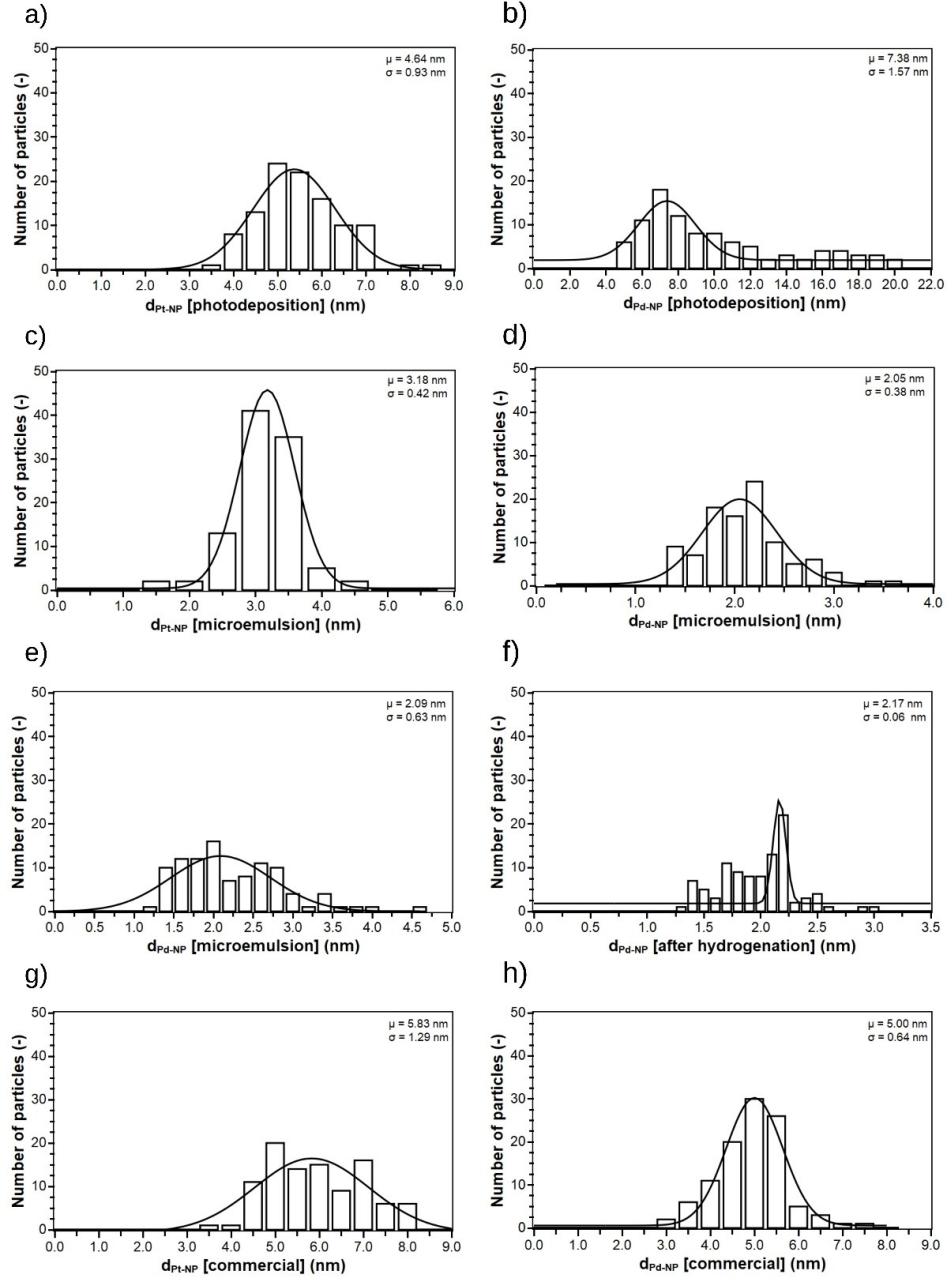
Size distribution of Pt and Pd NPs of PDPt (a), PDPd (b), MEPt (c), MEPd1 (d), MEPd2 (e), after the hydrogenation with MEPd2 (f), Pt@Al_2_O_3_ (g), and Pd@Al_2_O_3_ (h).

The size distributions of Pd and Pt NPs were obtained manually by measuring several NPs in TEM images. At least 100 particles were measured. The particle size distributions are shown in Figure 3. The particle distributions were fitted using a Gaussian fit to determine the average particle size μ and the standard deviation of the particle size σ. The values are given in Table 2. The variance of the Gaussian fit corresponds to the actual width of the distribution except for PDPd and MEPd2 after hydrogenation. Some large Pd crystals are found in the TEM images, but the largest number of particles is still within the small particle sizes. PDPt^[26]^ and PDPd prepared by photodepositon from the precursors H_2_PtCl_6_ and K_2_PdCl_6_ show larger average particle sizes of respectively 4.6 and 7.4 nm and broader variances of respectively 0.9 nm and 1.6 nm than the microemulsion‐prepared MEPt, MEPd1, and MEPd2. The irradiation time necessary for preparing PDPd of eight and a half hours led to larger particles than for PDPt, which had a shorter irradiation time of 30 min. No literature with NP size prepared with the photodeposition method was found. H_2_PtCl_6_ and K_2_PdCl_6_ were used as the precursor for MEPt and MEPd1, respectively, and PdCl_2_ for MEPd2. After adding the reductant ascorbic acid, MEPt was stirred at 55 °C for 60 min, MEPd1 similarly at 60 °C for 40 min, and MEPd2 was stirred overnight at 25 °C. Although different precursors and reaction conditions were used, MEPd1 and MEPd2 show similar variances and the same average particle size (2.1 nm). The average particle size of 3.2 nm MEPt is slightly larger than for MEPd1 and MEPd2. Similar Pt particle sizes with a similar procedure were found by Parapat et al.,^[28]^ showing that the average particle size and variance do not change using cellulose support instead of Al_2_O_3_. The microemulsion‐prepared catalysts show narrow size variances of 0.4–0.6 nm, which was reported before.[^3^, ^28^, ^29^] Commercially available Pt@Al_2_O_3_ shows an average particle size of 5.8 nm and a size variance of 1.3 nm, larger than any Pt@ModCe catalyst. The commercially available Pd@Al_2_O_3_ shows an average particle size of 5.0 nm and a size variance of 0.6 nm. Regarding Pd catalysts, only microemulsion‐prepared Pd@ModCe has a smaller particle size and similar variance than Pd@Al_2_O_3_.The considerations of this section show the sensitivity of average size from the deposition method. The size variation is less sensitive to the procedure variant of the method. The average particle size of MEPd2 does not change much after hydrogenation. The distribution of particle sizes becomes narrow, but it does not match the variance of the Gaussian fit at all. The particles with a size higher than 2.5 nm seem to have disappeared after catalysts use in hydrogenation, which was probably caused by leaching and agglomeration.

**Table 2:** Overview of the measured reciprocal lattice value 1/d, the calculated lattice plane distance d, and the determined Miller indices hkl for the Pt catalysts.

Catalyst	1/d (nm^−1^)	d (Å)	Δd (Å)	hkl
	4.41	2.27	0.15	(111)
PDPt	5.11	1.96	0.09	(200)
	7.23	1.38	0.08	(220)
	4.30	2.33	0.25	(111)
MEPt	5.00	2.00	0.20	(200)
	7.05	1.42	0.10	(220)
	4.43	2.26	0.10	(111)
Pt@Al_2_O_3_	5.06	1.98	0.12	(200)
	7.30	1.37	0.04	(220)

### Crystallinity

**Figure 4:**
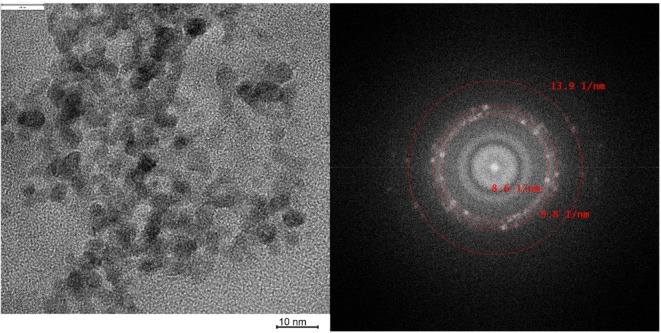
HR‐TEM from PDPt2 and the corresponding generated diffractogram.

The kind of crystallinity of Pd and Pt NPs was investigated by determining the Miller indices of the lattice planes through the Fourier transform of HR‐TEM images for PDPd, MEPt, MEPd1, MEPd2 (Figure 4) or directly recorded diffraction patterns for PDPt, Pt@Al_2_O_3_, and Pd@Al_2_O_3_. The software Digital Micrograph (Gatan) was used for Fourier Transform and evaluation, by measuring the ring diameter manually in 1/d. The lattice plane distances are characteristic values for each crystal composition and were compared and determined by the use of PCPDFWIN database (JCPDS 46–1043 and 04–0802). All values are listed in Tables 3 and 4. For all Pd and Pt crystals, at least the two lattice planes (111) and (200), and sometimes additionally (220) were identified, higher order lattice planes could not be evaluated due to the image‐resolution limit of the microscope and within the limited information in the Fourier transform. In the diffraction patterns of Pt@Al_2_O_3_ and Pd@Al_2_O_3_, the reflexes of aluminum oxide can overlay the reflexes of Pt or Pd because the lattice plane distances are similar. However, since no other typical reflexes of Pt and Pd are found, the same lattice plane distances can be assumed. The combination of the Miller indices of the lattice planes indicates a face‐centered cubic lattice structure (fcc) of Pt and Pd nanocrystals, which is also the characteristic lattice structure of Pt and Pd.[^33^, ^34^] Consequently, the deposition method does not influence the crystallinity when Pd or Pt NPs are formed.

**Table 3:** Overview of the measured reciprocal lattice value 1/d, the calculated lattice plane distance d, and the determined Miller indices hkl for the Pd catalysts.

Catalyst	1/d (nm^−1^)	d (Å)	Δd (Å)	hkl
PDPd	4.45	2.25	0.16	(111)
	4.95	2.02	0.16	(200)
	4.23	2.37	0.14	(111)
MEPd1	4.80	2.08	0.13	(200)
	6.95	1.44	0.04	(220)
	4.30	2.33	0.17	(111)
MEPd2	4.90	2.04	0.15	(200)
	6.95	1.44	0.08	(220)
After hydrogenation with MEPd2	4.35	2.30	0.13	(111)
	4.29	2.33	0.14	(111)
Pd@Al_2_O_3_	5.16	1.94	0.10	(200)
	7.20	1.39	0.05	(220)

**Table 4:** TON and TOF values for investigated catalysts.

Catalyst	TON_X=10 %_ (−)	TOF_X=10 %_ (h^−1^)	TON_X=50 %_ (−)	TOF_X=50 %_ (h^−1^)	t_1/2_ (min)	TON_t=1 h_ (−)	TOF_t=1 h_ (h^−1^)
Pt@Al_2_O_3_	165	3781	825	1559	31.8	1194	1194
PDPt	359	8677	1794	4815	22.4	2798	2798
MEPt	403	10020	2013	6180	19.5	3620	3620
Pd@Al_2_O_3_	89	3022	446	2526	10.6	891	891
PDPd	129	2545	643	1640	23.5	1235	1235
MEPd1^[a]^	132	5165	662	3751	10.6	1324	1324
MEPd2^[b]^	120	5440	600	2355	15.3	1200	1200
MEPd1^[a]^	333	6858	1667	3827	26.1	2668	2668

[a] K_2_PdCl_6_ as the precursor; [b] PdCl_2_ as the precursor; n_A0_ (ALB)=8.5 mmol; ΔTOF±10 %.

### EDX Mappings

**Figure 5:**
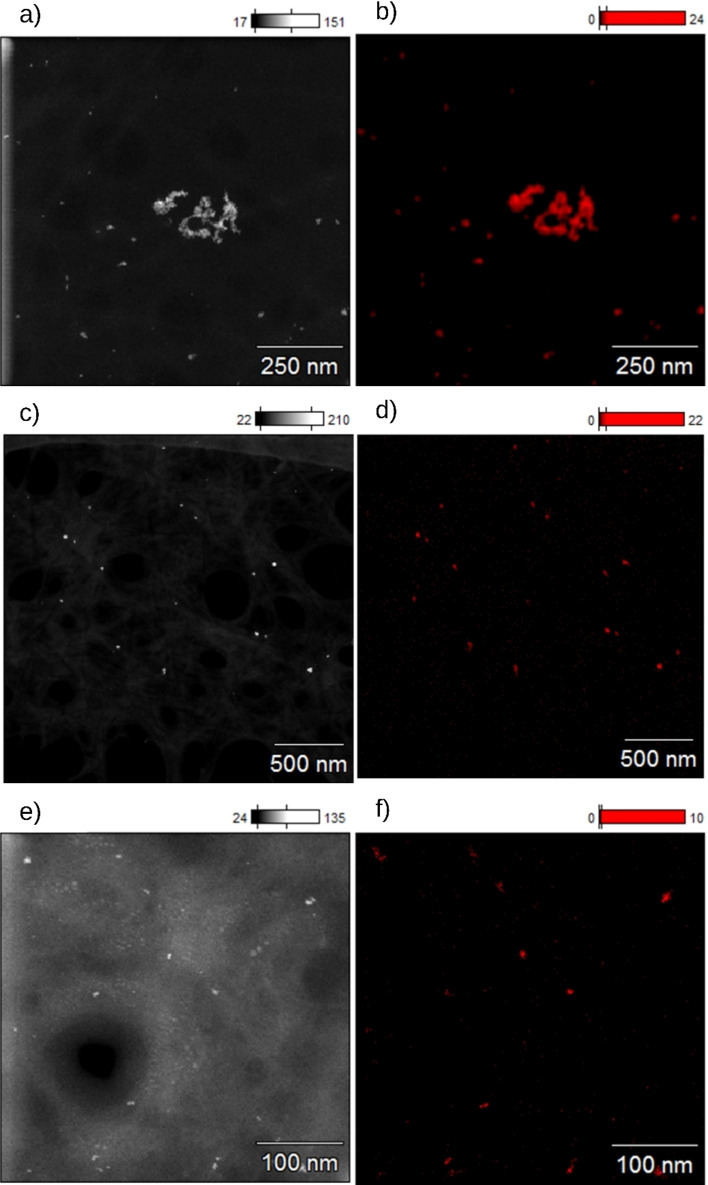
EDX mapping of PDPt (a and b), PDPd (c and d), and MEPt (e and f), with corresponding HAADF‐STEM images (left) and the EDS signals of Pt and Pd (right).

**Figure 6:**
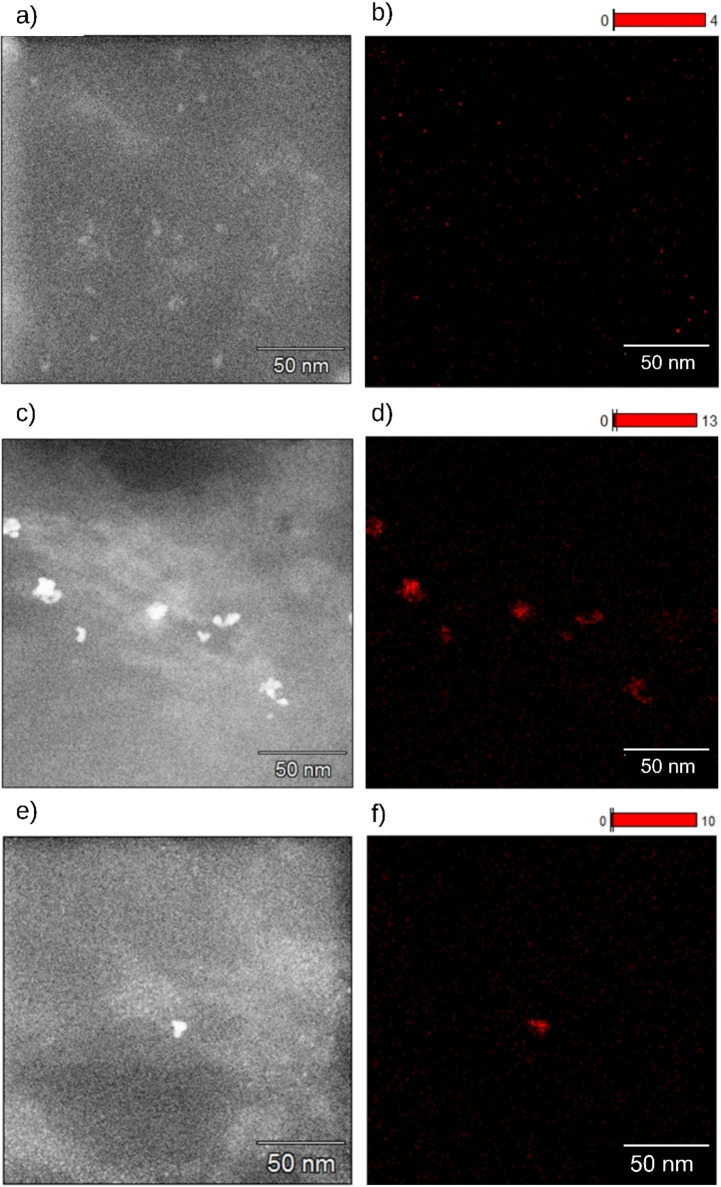
EDX mapping of MEPd1 (a and b), MEPd2 (c and d), and MEPd2 after the hydrogenation experiment with MEPd2 (e and f) with corresponding HAADF‐STEM images (left) and the EDX signals of Pt and Pd (right).

The manner of dispersion of Pt and Pd NPs on the cellulose support was intended to be discussed by mapping the EDX signals of Pt and Pd (Figures 5 and 6) and the respective spectra of the excerpts (see Figures S2–S14 in #ESI). Small particle sizes (approxm. 1–5 nm) at low loadings are difficult to detect via EDX mapping because of a relatively low number of involved atoms of each particle and thus a weak X‐ray yield and weak SNR. Due to long integration times of the EDX spectra, the Pt signals and Pd signals were clearly distinguishable from the background noise for the catalysts PDPt (Figures S2 and S3 in #ESI), PDPd (Figures S4 and S5 in #ESI), MEPt (Figures S6–S8 in #ESI), and MEPd2 (Figures S11 and S12 in #ESI). In PDPt (Figure 5a and b), large agglomerations of Pt particles of 20–100 nm are visible, caused by their high loading, which probably promotes the formation of agglomerations. The larger signals in PDPd are not agglomerations but regularly distributed nanocrystals of 10–20 nm, as shown in Figure 5c and d and discussed in the previous section. Small regularly distributed agglomerations of 10 nm are visible in MEPt. Larger agglomerations of 10–20 nm were also found in MEPd2 before hydrogenation. However, for MEPd1 and MEPd2 after hydrogenation any Pd Signal is weak and strongly overlapped with other signals and therefore could not clearly be distinguished from the background noise in the spectrum (Figures S9, S10, S13 and S14 in #ESI). So, in the mappings in Figure 6b only the background noise is visible while the other mappings show a few individual nanoparticles. As already described, particles can be hard to detect in EDX due to the small loading and the small particle size resulting in low intensities for the Pd signals in the EDX spectrum which can be easily overlapped by signals of other trace elements. Leaching might also affect the discoverability of particles. Nevertheless, due to the large difference of the atomic numbers, the contrast is strong enough to identify the Pd and Pt nanoparticles from carbon in the HR‐TEM and HAADF‐STEM. EDX mappings and additional diffraction patterns showed that crystalline particles were discovered for the catalyst PDPd and particle agglomerations for the catalysts PDPt, MEPt, and MEPd2.

### Hydrogenation of Allylbenzene

The hydrogenation of ALB was carried out as described in the experimental part. The hydrogen consumption V_H2_ was measured and converted into the ALB conversion X from which the initial reaction rate r_0_ and the activity A were calculated.^[35]^ Data evaluation is explained detail in Chapter 3 # ESI. In the following, the impact of different operating parameters and catalyst preparation methods are discussed based on r_0_ and A.

### Variation of Catalyst Concentration, ALB Concentration, and Stirrer Speed

**Figure 7:**
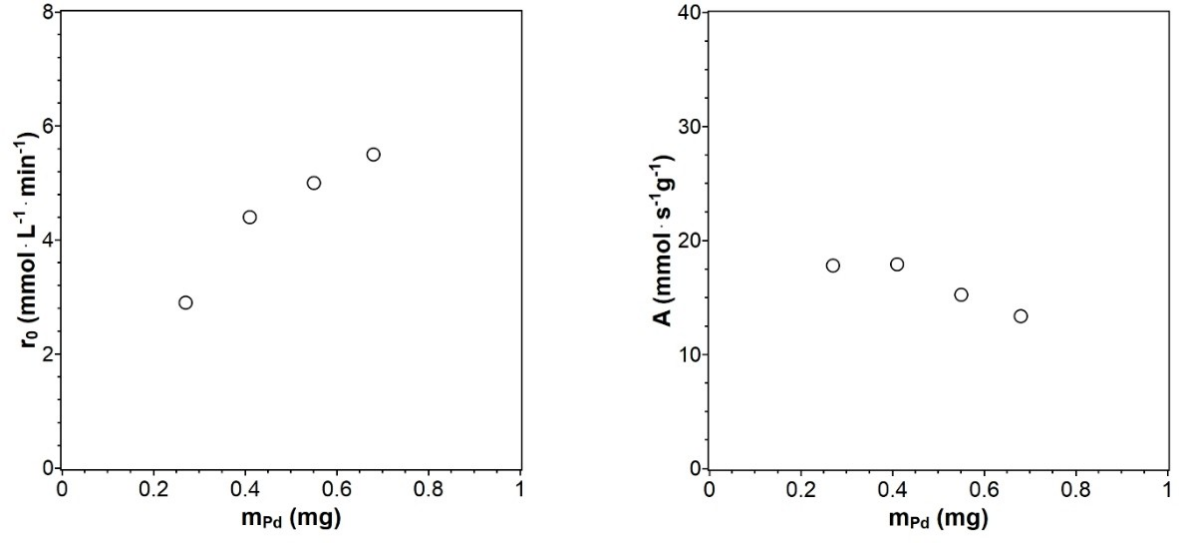
Initial reaction rate r_0_ (left) and activity (right) for different MEPd1 amounts (n=1200 rpm, V=100 mL, T=25 °C, p=1.1 bar, m_ALB_=1 g).

Hydrogenation reactions were performed, the concentration of the catalyst (Figure S17a in #ESI), changing the concentration of ALB (Figure S17b in #ESI), and the stirrer speed (Figure S18 in #ESI). The results were used to establish standard conditions for catalyst testing. Increasing the catalyst concentrations of MEPd1 shows an improvement in the initial reaction rate. Until a catalyst concentration of 150 mg (0.6 mg Pd), there seems to be an optimal use of the catalyst as the activity remains almost constant (Figure 7, right). For 100 mg and 150 mg of MEPd1, the ratio of the catalyst‐ALB amount is optimal in terms of the complete occupation and use of the active catalytic centers. The catalyst‐ALB ratio is not optimal when 200 mg and 250 mg of MEPd1 are used. For higher catalyst concentrations the rate increases only slightly and tends to reach a maximum equilibrium value, but activity continuously drops. The straightening of the conversion profile indicates that the reaction order changes from a pseudo‐first‐order reaction to a pseudo‐zero‐order reaction. When decreasing the ALB concentration (Figure S17b in #ESI), the initial slope and therefore the initial reaction rate Increases. Langmuir behaviour is expected, where r_0_ increases as long as free active sites are available for ALB and hydrogen adsorption.

**Figure 8:**
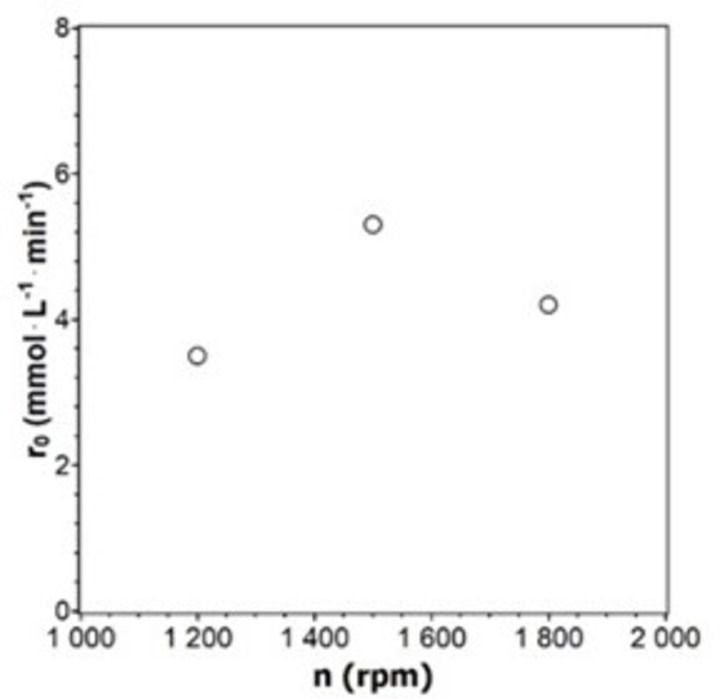
Initial reaction rates r_0_ for hydrogenation experiments with different stirrer speeds (100 mg MEPt, V=100 mL, T=25 °C, p=1.1 bar).

Three different stirrer speeds were investigated as a test for gas‐liquid mass transport limitations, using 100 mg of MEPt and 1 g of ALB (Figure 8). The reaction rate increases when the stirrer speed is raised to 1500 rpm because hydrogen bubbles become smaller and better dispersed, providing a larger interfacial area. The reaction speed decreases when the stirrer speed is raised further to 1800 rpm, because the used baffles started to move in the same direction as the stirrer.

The experimental parameters for catalyst testing were established from these first measurements to be 100 mg catalyst, 1 g ALB, and a stirrer speed of 1200 rpm (V=0.1 L). The catalyst is optimally utilized at this catalyst‐ALB ratio, and the stirrer speed could be increased further if necessary.

### Catalyst Testing

The Pt@ModCe catalysts PDPt and MEPt and the Pd@ModCe catalysts PDPd, MEPd1, and MEPd 2 were tested for the catalytic hydrogenation reaction. 100 mg of catalyst (except for 17 mg for PDPt and once 250 mg for MEPd1) and 1 g of ALB were used. The commercially available catalysts Pt@Al_2_O_3_ and Pd@Al_2_O_3_ were used as references. This investigation should also show the impact of the synthesis method on the catalytic activity. The analysis of the prepared catalysts, as discussed in Section 3.1, clearly shows catalysts with different sizes, size distributions, and loadings. All catalysts are catalytically active, as shown with the conversion profiles in Figure S19 in #ESI. The conversion profiles of all catalysts except MEPd2 and Pd@Al_2_O_3_ are not straight, meaning the reaction rates slow down with time. The strong influence of the preparation on catalyst performance is already apparent based on the conversion profiles. The catalysts prepared by the microemulsion method show faster ALB conversions than photodeposition‐prepared catalysts. The Pt‐catalyzed ALB conversion with the photodeposition‐prepared PDPt and microemulsion‐prepared MEPt catalysts proceed faster than the reference catalyst Pt@Al_2_O_3_. Regarding the Pd catalyzed conversion of ALB, the microemulsion prepared MEPd2 with PdCl_2_ as precursor proceeds with a similar rate as the reference Pd@Al_2_O_3_. The third‐fastest conversion is with MEPd1 (K_2_PdCl_6_ as a precursor), followed by the photodeposition‐prepared PDPd.

**Figure 9:**
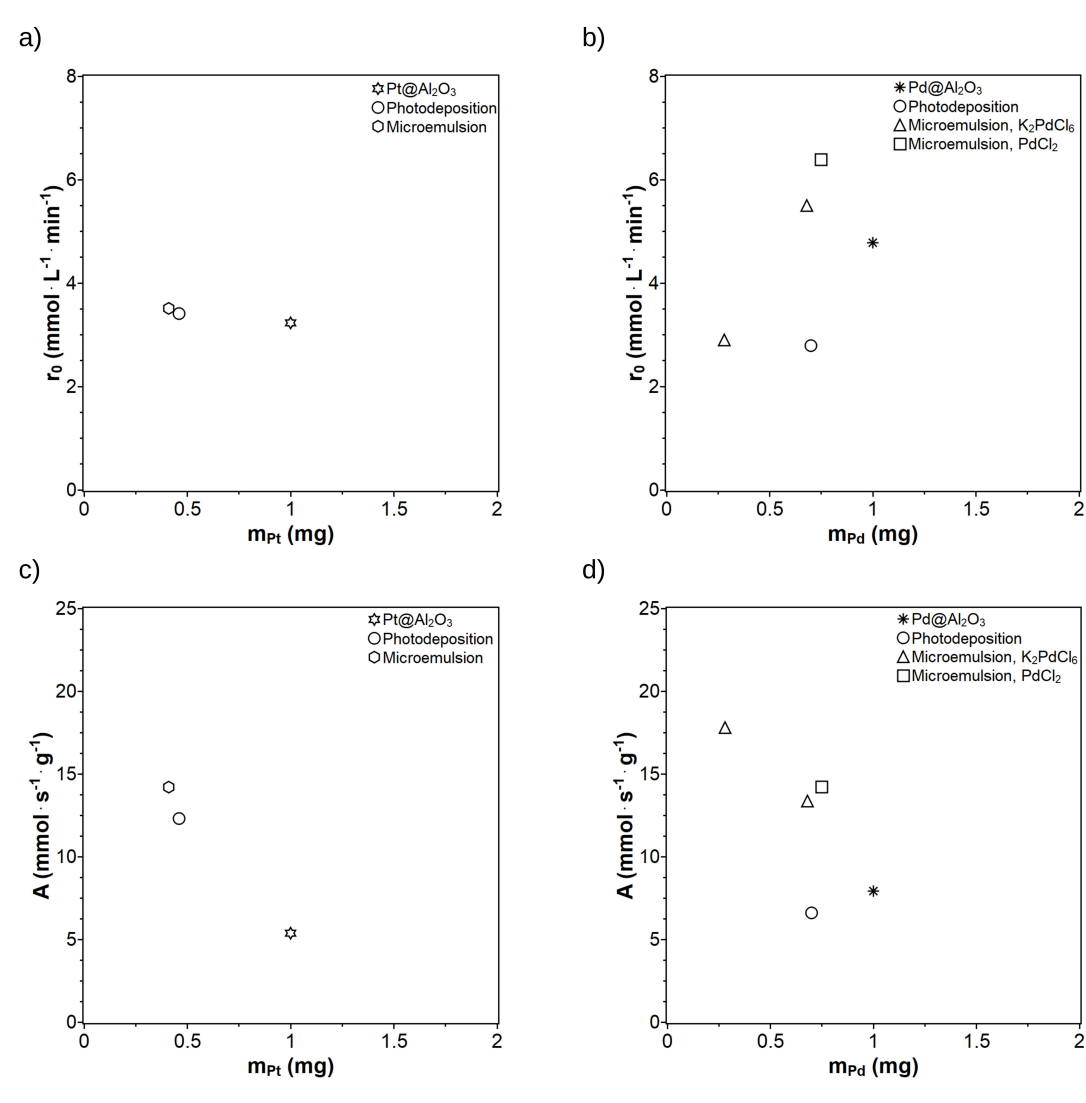
Initial reaction rate r_0_ (top) and catalyst activity A (bottom) against the mass of Pt (a and c) and Pd (b and d) nanoparticles deposited via Photodeposition (PDPt and PDPd), microemulsion deposition (MEPt, MEPd1, and MEPd2), and Pt@Al_2_O_3_ and Pd@Al_2_O_3_ as references. K_2_PdCl_6_ was the precursor for MEPd1 and PdCl_2_ for MEPd2.

The initial reaction rates r_0_ and the catalyst activities were plotted against the mass of the metal NPs m_Pt_ and m_Pd_ (Figure 9). Different catalytic active metal amounts were present since the same masses of Pt@ModCe and Pd@ModCe with different loadings were used for hydrogenation. Therefore, only the catalyst activities with similar metal amounts in the reaction system are directly comparable. In Figure 9a, all Pt@ModCe catalysts show similar initial reaction rates to the Pt@Al_2_O_3_ despite the lower Pt amount. That is caused by the preparation method and the smaller NP sizes of the synthesized Pt catalysts. The activity for almost the same Pt concentration could not be compared due to the different metal masses. However, from the progress for Pd, it is expected that for the same amount of metal, the cellulose‐supported catalysts are more active. Whether MEPt or PDPt is the better catalyst cannot be decided based on the available data. Perhaps the influence of the particle size would become better visible if greater catalyst concentrations were used. The data points from the variation of the catalyst mass at 100 mg and 250 mg MEPd1 from Figure 7 in the previous chapter are also shown in the reaction rate and activity plots for the Pd@ModCe catalysts in Figure 9. The microemulsion‐prepared catalyst MEPd1 shows an initial reaction rate similar to PDPd with only half of their Pd mass. The initial reaction rate using 250 mg of MEPd1 is the second‐highest rate after MEPd2. The catalysts MEPd1 (250 mg), MEPd2, and PDPd have almost the same Pd amount, so their performance is directly comparable. The microemulsion method prepared MEPd1 and MEPd2 show the highest reaction rates. This difference cannot be explained by the small mass differences as a single reason and, therefore, is directly related to the preparation process and the characteristics of the Pd NPs. The average NP sizes of MEPd1 and MEPd2 with (2.1±0.6) nm and (2.1±0.4) nm, respectively, are significantly smaller than the particle sizes of other Pd catalysts. The average Pd particle sizes for Pd@Al_2_O_3_ and PDPd are (5.0±0.6) nm and (7.4±1.6) nm, respectively. Additionally, the microemulsion deposited NPs are evenly dispersed on the ModCe surface. Despite Pt being the generally less active metal than Pd, Pt@ModCe shows partially higher reaction rates than Pd@ModCe and generally has similar activities. Microemulsion‐prepared catalysts are more active toward the ALB hydrogenation than catalysts prepared via photodeposition and the reference catalysts.

Two publications with similar approaches were found. In König et al., 2 g of ALB in methanol at 25 °C and 1.5 mg catalyst (30 wt %, 0.45 mg Pt) were used. The reaction rate was evaluated in the same manner as in this work. The reaction rate is 2.3 mmol L^−1^ min^−1^, and the calculated activity is 3.8 mmol g^−1^ s^−1^_._ ^[8]^ This approach is comparable to the experiment with 2 g ALB and 100 mg MEPt (0.4 mg Pt) from Section 3.4.2, where the reaction rate was 3.3 mmol L^−1^ min^−1^ and the activity 13.5 mmol g^−1^ s^−1^. MEPt is more active than the microemulsion deposited Pt NPs on ethoxylated polyethylene imine. In the work of Oliveira et al., 1 g ALB in methanol at 35 °C and 35 mg catalyst (2 wt %, 0.7 mg Pd) were used, which is comparable with the Pd@ModCe experiments.^[36]^ The authors have only shown the conversion profiles in their manuscript, but from the slope, an initial reaction rate of 1.69 mmol L^−1^ min^−1^ and a corresponding activity of 2.41 mmol g^−1^ s^−1^ was estimated, which is lower than the activity of any tested cellulose catalyst.

For a better comparison of the methods used to deposit either Pt or Pd nanoparticles on cellulose, TON and TOF have been calculated for all catalysts shown in Figure 9. The results are summarized in Table 4. The calculation was carried out for three different points within the reaction progress. The first TON/TOF pair was calculated from the initial reaction rate r_0_ for an ALB conversion of 10 %. The second TON/TOF pair was calculated for an ALB conversion of 50 %. The reaction half‐life t_1/2_ can also be specified for a 50 % conversion. The last TON/TOF pair was calculated for a reaction time of 1 hour. The TON and TOF values are based on the molar quantities rather than on mass which were used to calculate the activity.

As expected, with increasing ALB conversion, TON values increase and TOF values decrease. Even based on these values, the two commercial catalysts used as reference show the weakest performance among the investigated systems. For both metals, the supported catalysts prepared using the microemulsion method show the best performance. The TOF values are about 3600 h^−1^ and 2700 h^−1^ for MEPt and MEPd1*, respectively. Although the fastest reaction obviously proceeds with the commercial Pd@Al_2_O_3_ catalyst (t_1/2_ about 11 min, see also Figure S19 in #ESI), the TOF value is the lowest. Usually, catalytic performance is compared for the same TON value, but the metal loading of the prepare samples is quite different (between 0.2 and 1.0 wt %). The commercial catalysts have the highest metal loading that's why among the Pd catalysts, Pd@Al_2_O_3_ is much faster and half‐life is the lowest. However, the Pd nanoparticles in Pd@Al_2_O_3_ are less active due to their larges particles sizes and nanoparticle agglomeration. The cellulose‐supported catalysts have much lower loadings, but the smaller nanoparticles combined with a better dispersion lead to an increased activity. In summary, all prepared catalysts show a better performance than the commercial ones, but for catalyst recycling also its stability is important.

### Catalyst Stability

In this work, metal nanoparticles are immobilized on cellulose. To investigate the stability of the catalysts, the metal loading after its use for hydrogenation of ALB was determined to calculate the metal leaching for Pd@ModCe and Pt@ModCe catalysts. The NPs of all catalysts were dissolved in aqua regia three months after the experiments. Three Pd and three Pt catalysts have been selected to determine the metal content in the samples. Samples were used under standard reaction conditions (1 g ALB, 100 mg catalyst). All loadings of the catalysts before and after hydrogenation are listed in Table 5. The catalyst loadings do not differ for MEPt after hydrogenation. However, they differ significantly for MEPd1 and MEPd2 after hydrogenation by 33 %, 66 %, and 47 %, respectively. So, no leaching occurs with Pt@ModCe catalysts even after three months of storage of the remaining reaction suspension. However, leaching takes place for the Pd@ModCe catalysts. The sole possible explanation for these observations is a good Pt immobilization on ModCe, whereas Pd NPs on ModCe are unstable. The intermolecular interactions between Pt NPs and ModCe are probably stronger than between Pd NPs and ModCe. The stability of immobilization is characterized by electrostatic interactions using the zeta potential. The zeta potential is the electric potential at the boundary of the stern double layer and is thus the total charge on the surface of NPs and depends on pH value. The stern double layer is the layer of counter ions on the NP surface, attracted by the loading of this surface.^[37]^ Opposite‐loaded surface charges of NPs and support indicate a stable immobilization. The zeta potential of cellulose NPs is about −60 mV.^[38]^ The published zeta potential values of Pt and Pd NPs are diverse, especially the measured pH values that are often not given. Our findings regarding the superior stability of Pt nanoparticles (Pt‐NPs) in comparison to Pd nanoparticles (Pd‐NPs) resonate with prior investigations into the biosorption of these metals. Although the Zeta potential might not be the predominant determinant, existing evidence indicates that the interplay between nanoparticles and functional groups present on the cellulose substrate exerts substantial influence. Xu et al. have previously discussed the competitive biosorption dynamics of Pt(IV) and Pd(II), underscoring the relevance of such interactions.^[39]^ This study showed the difference of biosorption mechanisms of Pt(IV) and Pd(II) on P. vermicola as the biosorbent. The study also revealed that different functional groups played crucial roles in adsorbing Pt and Pd. They reported that Pt ions have a higher affinity for the bacterial biomass compared to Pd ions. Although this study deals with ionic forms of the metals, it suggests a stronger interaction between Pt and the functional groups present on the bacterial cell wall, which could translate to similar behavior with Pt‐NPs and cellulose, a type of complex carbohydrate with various functional groups.

**Table 5:** Determined catalyst loading before and after the hydrogenation experiments.

Catalyst	Loading before experiment (wt %)	Loading after experiment (wt %)
MEPd1	0.3	0.2
MEPd1	0.3	0.1
MEPd2	0.7	0.4
MEPt	0.4	0.4
MEPt	0.4	0.4
MEPt	0.4	0.4

The interactions between metal nanoparticles and cellulose nanofibrils also has been reported. It highlights the involvement of different functional groups on the cellulose surface, such as hydroxyl, carboxyl, and carbonyl groups, in binding metal nanoparticles through various mechanisms like electrostatic interactions, hydrogen bonding, and covalent bonding.^[40]^ This strengthens the argument that interaction with these functional groups could contribute to the observed stability difference between Pt‐NPs and Pd‐NPs on the cellulose. Therefore, the higher stability of Pt NPs can be attributed to their greater affinity for hydroxyl, carboxyl, and carbonyl groups on the cellulose surface compared to Pd NPs. The high reaction rate and activities of the microemulsion‐prepared Pd@ModCe catalysts are probably partly caused by leached Pd NPs.

### Catalyst Recycling

**Figure 10:**
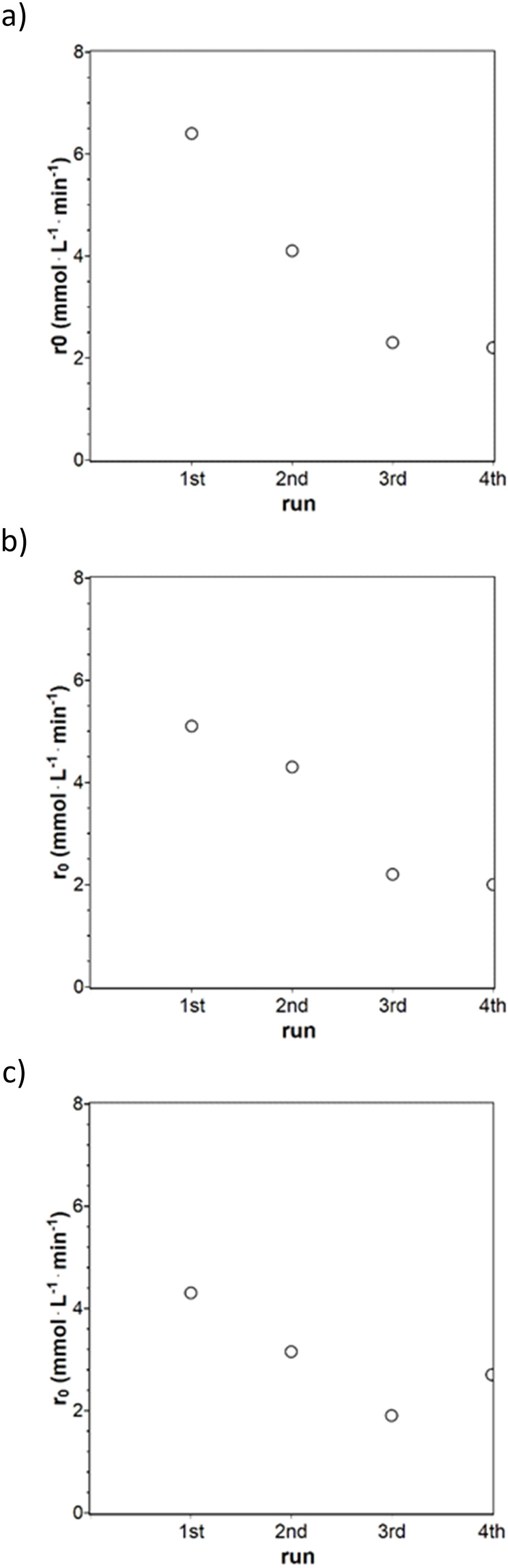
Initial reaction rates for the recycling of MEPd2 (a, 100 mg, 1 g ALB), PDPt (b, 100 mg, 110 mg ALB), and Pd@Al_2_O_3_ (c, 100 mg, 1 g ALB). PDPt, and Pd@Al_2_O_3_ were reloaded with ALB, and MEPd2 was washed and dried before reuse (n=1200 rpm, V=100 mL, T=25 °C, p=1.1 bar).

Recycling tests of metal NPs immobilized on cellulose were conducted for the catalysts MEPd2, PDPt, and Pd@Al_2_O_3_ (Figure 10) to evaluate the general recyclability of the cellulose‐supported catalysts compared to a commercially available catalyst. Each recycling experiment was conducted with 100 mg of catalyst and 1 g of ALB for MEPd2 (0.7 mg Pd) and Pd@Al_2_O_3_ (1 mg Pd) and PDPt (2.7 mg Pt) with 100 mg ALB. The recycling tests were done first with MEPd2 (Figures 10a and S20a), separated after each recycling reaction. The whole catalyst was not retained due to handling losses, resulting in the loss of catalyst material and the change of the catalyst‐ALB ratio. The catalyst mass for the second run was 93 mg, 80 mg for the third run, and 71 mg for the fourth run, and the conversion profile and the initial reaction rate decreased significantly with each further recycling. The profiles become curvier with each recycling. That was due to the activity loss, and a smaller catalyst‐ALB ratio leads to lower reaction rates. The previous chapter shows that the Pd@ModCe catalyst leaching causes lower loadings with each run. The findings from the size distribution, TEM of MEPd2 after one hydrogenation experiment in chapters 3.1 concluded that agglomeration of smaller NPs occurs. The last two initial reaction rates are the same despite the lower catalyst amount, indicating that the leaching and agglomeration of particles are limited. Leachable particles were probably washed out. The recycling tests with Pd@Al_2_O_3_ (Figures 10c and S20c) were conducted by leaving the reaction suspension in the setup and adding only new ALB for the next run. The resulting conversion profiles were straight, indicating a pseudo‐zero‐order reaction. However, the conversion profiles and initial reaction rates decrease similarly to the Pd@ModCe catalysts. The recycling tests with PDPt (Figures 10b and S20b) were carried out similar manner to Pd@Al_2_O_3_. The concentration profiles were straight for the first two runs, indicating a zero‐order reaction and becoming curvier for the third and fourth runs. Like in the recycling tests for MEPd2 and Pd@Al_2_O_3_, in PDPt recycling, the reaction rate decreases with each run. The initial reaction rates decrease independently from the support with each recycling, which is probably related, on the one hand, to the competitive adsorption of ALB and PB at the Pt and Pd NPs. Since agglomerations were found with TEM recordings and EDX mapping after an experiment with MEPd2, the decrease of the reaction rate, on the other hand, is also probably caused by agglomerated particles on the surfaces of all catalysts for the most part and also by leaching. The conversion profiles for the Pd@Al_2_O_3_ remain straight, as the profiles for MEPd2 and PDPt get curvier with each run. Even by applying a high catalyst ALB ratio, the PDPt loses activity at the end of the reaction, causing a rounder profile. The curving of the profiles is probably caused by the cellulose support. The hydrophilic surface of ModCe probably makes the adsorption of the hydrophobic ALB and PB harder, and the cellulose NPs surround the metal NPs. That would limit the space for the diffusion of the reactants and slow down the reaction after a certain PB concentration is reached.

The TON and TOF values have been calculated for the initial reaction phase (TON‐10/TOF‐10) and for the time, when the 1^st^ run for each catalyst reaches maximum conversion (TON_x_/TOF_x_). This allows to compare the performance of the consecutive runs directly with the first one. The results are summarized in Table 6.

**Table 6:** TON and TOF values for recycling experiment.

Catalyst	Run	TON‐10 (−)	TOF‐10 (h^−1^)	X (−)	TON_X_ (−)	TOF_X_^[a]^ (h^−1^)
	1	41	14924	0.98	404	5392
	2	41	12583	0.90	371	4952
PDPt^[b]^	3	41	6438	0.80	330	4402
	4	41	5852	0.60	248	3301
	1	129	5829	1.00	1286	3859
	2	138	4061	0.58	802	2407
MEPd2^[c]^	3	161	2680	0.39	627	1881
	4	181	2762	0.30	544	1631
	1	90	2746	0.97	873	2096
	2	90	2011	0.64	576	1383
Pd@Al_2_O_3_	3	90	1213	0.55	495	1189
	4	90	1724	0.50	450	1081

[a] calculated for the time, when the 1^st^ run reaches maximum conversion (4.5 min for PDPt, 20 min for MEPd2, and 25 min for Pd@AL_2_O_3_); [b] 100 mg ALB; [c] catalyst was washed and dried between the runs; n_A0_ (ALB)=8.5 mmol (0.5 mmol for PDPt); ΔTOF±10 %.

As shown for the initial reaction rates, the activity decreases from run 1 to run 3 but stabilizes at run 4. This is also obvious from TOF‐10 and TOF_x_. Due to the lower ALB concentration used in the case of the PDPt catalyst, almost complete conversion is achieved after 4.5 min. For MEPd2 and Pd@Al_2_O_3_, the maximum conversion for the first run is reached after 20 min and 25 min, respectively. The TON and TOF values are the lowest for the commercial Pd@Al_2_O_3_ catalyst and highest for the PDPt catalyst, because of a lower amount of ALB. Among the Pd catalysts, MEPd2 shows better catalytic activity. As mentioned earlier, leaching of metal nanoparticles into solution or agglomeration of metal nanoparticles could be a possible reason for the lower activity. This could apply to the catalysts produced, especially MEPd2. However, the commercially available catalyst, which was not removed from the reaction solution between runs and is decorated with larger size metal nanoparticles, shows a significant decrease in TOF. The TOF dropped to 50 % after the fourth run, which is most likely not due to particle agglomeration or leaching. This could be due to the fact that there is no hydrogen in the reaction solution at the beginning of the hydrogenation reaction and the surface of the catalyst is covered with ALB, leading to a fast reaction. After completion of the reaction, the hydrogen is adsorbed on the metal surface when ALB is added for the next run. It is known that hydrogen has a strong affinity for platinum or palladium, which is why they are used as metals in hydrogenation reactions. From this point of view, the initial situation for the second, third and fourth run changes. There is still a certain decrease in activity, which could be due to adsorbed products accumulating in the reactor. Therefore, there is already a general trend in the activity based on how the experiments are done, and compared to the trend of the commercially available catalyst, the catalysts produced show a good performance. For the MEPd2 catalyst, which was removed between runs and used in lower quantities from run to run, it must be considered that the ratio between ALB and Pd increases. The ratio of substrate to catalyst has also a significant impact on the reaction.

## Conclusions

In this work, Pt and Pd NPs were successfully immobilized onto cellulose via photodeposition and microemulsion deposition, and compared to commercially available alumina‐supported catalysts regarding their characterization, initial reaction rate, catalyst activity, leaching and recyclability on the hydrogenation of allylbenzene. The characterization included the loading, size distribution, crystallinity, and dispersion of the particles on cellulose. The photodeposition of Pd NPs onto a non‐semiconductor was reported for the first time. The obtained metal loadings were in the range of 0.4–2.7 wt %. The comparison of synthesized catalysts with the literature on the respective deposition method showed that the loading yields of the photodeposition and microemulsion methods are not influenced by the support material. The size distribution was dependent on the NPs prepared by the microemulsion method and showed the smallest NP size (MEPt=3.2 nm, MEPd=2.1 nm) and a very even and narrow size distribution. The NPs prepared by photodeposition (PDPt=4.6 nm, PDPt=7.4 nm) and the NPs from the commercial catalysts (Pt@Al_2_O_3_=5.8 nm, Pd@Al_2_O_3_=5.0 nm) showed larger average particle sizes. The crystallinity of the NPs was shown to be independent of the deposition method and support material. Examples of regularly distributed particles or particle agglomerations were found for the catalysts prepared by photodeposition and MEPt. Mass transport limitations were revealed in varying the catalyst‐educt ratio. Testing the prepared catalysts for the allylbenzene hydrogenation showed the highest activity for microemulsion‐prepared catalysts followed by the photodeposition‐prepared and reference catalysts. However, the Pd NPs are not as well stabilized onto cellulose as Pt NPs exhibited by leaching. The recyclability of the cellulose‐supported catalysts is similar to the reference catalyst. The reaction rate decreases for all catalysts after each run, probably caused by particle agglomeration on the catalyst surface, leaching, and the competitive adsorption of allylbenzene, propyl benzene and hydrogen on the catalyst surface. However, the results show that cellulose can be used an alternative support material in catalytic reactions under mild reaction conditions.

## Experimental Part

### Chemicals

For the manufacture of modified cellulose (ModCe), unmodified cellulose (UnCe), isolated from *Styela Clava* (Otto‐Van‐den‐Berg‐Company), and sulfuric acid (H_2_SO_4_, 96 wt %, ≥99 %, Roth) were used. For the deposition of Pt and Pd NPs onto cellulose, ascorbic acid (AA, 99 %, Alfa Aesar), hexachloroplatinic acid solution (H_2_PtCl_6_, 8 wt % in H_2_O, Sigma Aldrich), potassium hexachloropalladate (K_2_PtCl_6_, >26.3 %, Alfa Aesar), Pd chloride (PdCl_2_, 99 %, Sigma Aldrich), cyclohexane (CH, ≥99.9 %, Roth), 1‐butanol (≥99 %, Roth), and Triton X‐100 (99 %, Roth) were applied. For hydrogenation experiments, allylbenzene (ALB, 98 %, Alfa Aesar), methanol (MeOH, ≥99.9 %, VWR), propylbenzene (PB, 98 %, Arcos Organics), trans‐β‐methyl styrene (TMS, 99 %, Sigma Aldrich), hydrogen (H_2_, 5.0, Air Liquide), Pt on alumina (Pt@Al_2_O_3_, 1 wt %, Alfa Aesar), and Pd on alumina (Pd@Al_2_O_3_, 1 wt %, Alfa Aesar) were used. Commercial Pt and Pd standard solutions (1000 mg/L, Sigma Aldrich) were used to calibrate the ICP spectrometer. Hydrogen chloride (HCl, 37 wt %, 99 %, Roth) and nitric acid (HNO_3_, 68 wt %, 99 %, Roth) were applied for ICP sample preparation. All chemicals were used as received.

### Isolation of Cellulose Nanomaterials

Modified cellulose (ModCe) synthesis by sulfuric acid hydrolysis was conducted by a method reported by van den Berg et al. with slight modifications.^[41]^ The synthesis was done in a double‐walled glass reactor (maximum volume 600 mL) and the temperature was controlled by a thermostat (F6 C25, Haake). Under vigorous stirring, concentrated H_2_SO_4_ (120 mL, 98 %) was slowly added to UnCe (4.5 g) that was dispersed in water (120 mL) at 4 °C. Then, the temperature was increased to 60 °C, where the solution was stirred for another 2 hours to accelerate the hydrolysis, before the suspension was cooling down to 4 °C to stop the reaction. The obtained suspension was filtered and washed over a small‐pore glass‐fritted filter until the pH became neutral. The residue was freeze‐dried (Alpha 1–4, Christ) for three days.

### Photodeposition of Metal NPs Onto ModCe

Pt@ModCe and Pd@ModCe catalysts were manufactured via photodeposition (PD) and are named as PDPt and PDPd, respectively. The preparation of PDPt was published recently.^[26]^ For photodeposition of Pd NPs onto ModCe, K_2_PdCl_6_ (8 mg) was placed in a round bottom flask, a ModCe dispersion (3.3 g/L, 60 mL, V_MeOH_/V_water_=70/30) was added, and the mixture was purged with nitrogen for 15 min. The dispersion was irradiated by a 300 W Xe‐lamp (Quantum Design Europe) placed in 10 cm distance using the full light spectrum. The irradiation of the dispersion was carried out for 510 min. The prepared catalyst was filtered, washed with water, and finally freeze‐dried.

### Deposition of Metal NPs Onto ModCe Via Microemulsion Method

One Pt@ModCe and two Pd@ModCe catalysts were manufactured via the microemulsion method and named as MEPt, MEPd1, and MEPd2, respectively. The deposition was carried out based on the work of Parapat et al.^[42]^ At first, two microemulsions, one containing the metal precursor (ME1) and one containing ascorbic acid as the reducing agent (ME2), were prepared. The microemulsions contained water, the co‐surfactant n‐butanol, the surfactant Triton X‐100, and cyclohexane with the mass ratios m_water_/m_n‐butanol_/m_Triton X‐100_/m_cyclohexane_=15/35/35/15. For the reaction, ME1 was placed into a double‐walled glass reactor together with the required amount of ModCe and ME2 was slowly added in a time span of 30 min at 25 °C. The suspension was stirred at 25 °C for 30–45 min and then heated up and stirred at the desired temperature. The mixture was then cooled down and then washed with water and acetone. The used amounts of microemulsions, ModCe, and precursor as well as the heating conditions are listed in Table 7. MEPt and MEPd1 were freeze‐dried and MEPd2 was washed with acetone and air‐dried overnight.

**Table 7:** The used amounts of microemulsions ME1 and ME2, ModCe, Precursors, stirring temperature with the corresponding time for deposition of Pd and Pt NPs by microemulsion on ModCe.

Catalyst	Precursor	m_ME1_ (g)	m_ModCe_ (g)	m_Precursor_ (mg)	m_ME2_ (g)	T (°C)	t (min)
MEPt	H_2_PtCl_6_	100	1	262.8	50	55	60
MEPd1	K_2_PdCl_6_	100	0.3	11.7	5	60	40
MEPd2	PdCl_2_	170	1	17.1	50	25	Overnight

### Catalyst Characterization

#### ICP‐OES

Pt and Pd loadings catalysts were determined from inductively coupled plasma atomic emission spectroscopy (ICP‐OES) using a Varian 715 ES spectrometer (Agilent Technologies). The setup calibration was done with standard solutions with concentrations of 2.5 ppm, 5 ppm, 10 ppm, 20 ppm, and 25 ppm.

The NPs from the catalysts were dissolved overnight by adding a mixture of HCl, HNO_3_, and H_2_SO_4_ (10 mL, V/V/V=3/1/1) to the sample (20 mg). After this time, ModCe was filtered off, and the solution was diluted with water up to 50 mL. 5 mL of this solution were taken and further diluted up to 15 mL. The catalyst loading was calculated from the prepared sample solution's measured Pt concentration.

#### S/TEM, Diffraction Pattern, and EDX‐Mapping

For investigation of the morphology, size distribution, crystallinity, and particle dispersion of the Pt and Pd NPs in the M@ModCe (M=Pt, Pd) catalysts, scanning/transmission electron microscopy (S/TEM) was conducted. The samples were prepared by dispersing 1 mg catalyst sample in 1 mL water. 3 μL of the dispersion were drop‐casted and air‐dried on 300 mesh holey carbon Cu‐grids. A conventional TECNAI G^2^20 TEM (FEI/TFS Company, USA, operated at 200 kV, with LaB6 electron emitter) and a probe cs‐corrected JEM‐ARM300F2 HR‐STEM (JEOL Ltd., Japan, operated at 300 kV, with cold FEG emitter) were used. For energy dispersive X‐ray spectroscopy mappings (EDX‐Mappings) the latter instrument is equipped with a windowless Dual SDD EDS system (JEOL Ltd.) with 2×158 mm^2^ and a detection angle of 2.2 sr. To distinguish NPs from support series of HAADF‐STEM images as well as conventional TEM BrightField images were acquired. For evaluating the diffraction patterns and determining the size distributions of Pd and Pt NPs by measuring the NP size in the TEM images, the image processing and acquisition software Digital Micrograph from Gatan Inc. (Version 3.43.3213.0) was used. The data analysis and visualization program, QTiPlot (Version 5.12.8) was used for fitting the size distribution.

### Hydrogenation Experiments

For the heterogeneously catalyzed hydrogenation reactions, a double‐walled reactor was loaded with ALB (1 g, 6.7 mmol) and a catalyst suspension (100 mL of MeOH, 100 mg catalyst, except 17 mg in the case of PDPt) was added. Hydrogen was mixed into the liquid phase using a gas dispersion stirrer. Baffles were used for a better dispersion of the the gas bubbles and to avoid trombe formation. The reactor was tempered at 25 °C by a thermostat (F6 C25, Haake). The stirrer was switched on at a speed of 1250 rpm, and then the reactor was evacuated and purged with nitrogen three times. Next, the stirrer speed was lowered to 800 rpm, and the suspension was kept at these conditions for 20 min to obtain the temperature equilibrium. Then, the stirrer speed was raised again to 1200 rpm, the reactor was evacuated, and the stirrer was stopped. The reactor was filled with hydrogen until a total pressure of 1.1 bar. The reaction was started when the stirrer was turned on again, mixing the hydrogen into the suspension. The reaction was done at constant pressure (isobaric) using a pressure controller (Bronckhorst) and consumed hydrogen was redosed as soon as the pressure drop was >0.2 %. The pressure and hydrogen consumption during the whole hydrogenation experiment were monitored by an Excel macro. After the reaction, a sample was taken and analyzed by gaschromatography.

### Gaschromatography

The samples from the hydrogenation of ALB were filtered through a cellulose acetate (CA, 0.2 μm) syringe filter and analyzed by gas chromatography (GC) using a chromatography setup from Shimadzu (model GC‐2010) equipped with a Supelcowax column from Supelco and a flame ionization detector (FID). The retention times were 4.7 min, 4.2 min, and 6.7 min for ALB, PB, and TMS.

## Funding

Tabea Angela Thiel and Michael Schwarze acknowledge the support funded by the Deutsche Forschungsgemeinschaft (DFG, German Research Foundation) under Germany's Excellence Strategy–EXC 2008/1 (UniSysCat)–390540038 and project number 403371556–GZ: INST 131/789‐1 FUGG.

## Author Contributions

T.A.T. designed the experiments, collected, analyzed as well as interpreted the data, and wrote the first draft of the manuscript. M.S. (1) provided the cellulose material for the experiments. M.S. (2) conceptualized and supervised the project. T.A.T., R.Y.P, M.S. (1), and M.S. (2) contributed to the text and contents, including discussions, revisions, and edits. All authors have reviewed the content and approved its publication.

## Conflict of Interests

The authors declare no conflict of interest.

1

## Supporting information

As a service to our authors and readers, this journal provides supporting information supplied by the authors. Such materials are peer reviewed and may be re‐organized for online delivery, but are not copy‐edited or typeset. Technical support issues arising from supporting information (other than missing files) should be addressed to the authors.

Supporting Information

## Data Availability

The data that support the findings of this study are available from the corresponding author upon reasonable request.
