# Supplementary material for: Catalytic Activity of Cellulose‐Supported Platinum and Palladium Nanoparticles for Allylbenzene Hydrogenation
Source: Chemistry. 2024 Dec 12;31(2):e202402952. doi: 10.1002/chem.202402952 (PMC11724257; doi:10.1002/chem.202402952)
Supplement: Supplementary file 1 — Supporting Information [file CHEM-31-e202402952-s001.pdf]

Chemistry–A European Journal

Supporting Information

Catalytic Activity of Cellulose-Supported Platinum and Palladium Nanoparticles for Allylbenzene Hydrogenation

Tabea Angela Thiel,* Riny Yolandha Parapat, Michael Schroeter, and Michael Schwarze*

1. TEM analysis

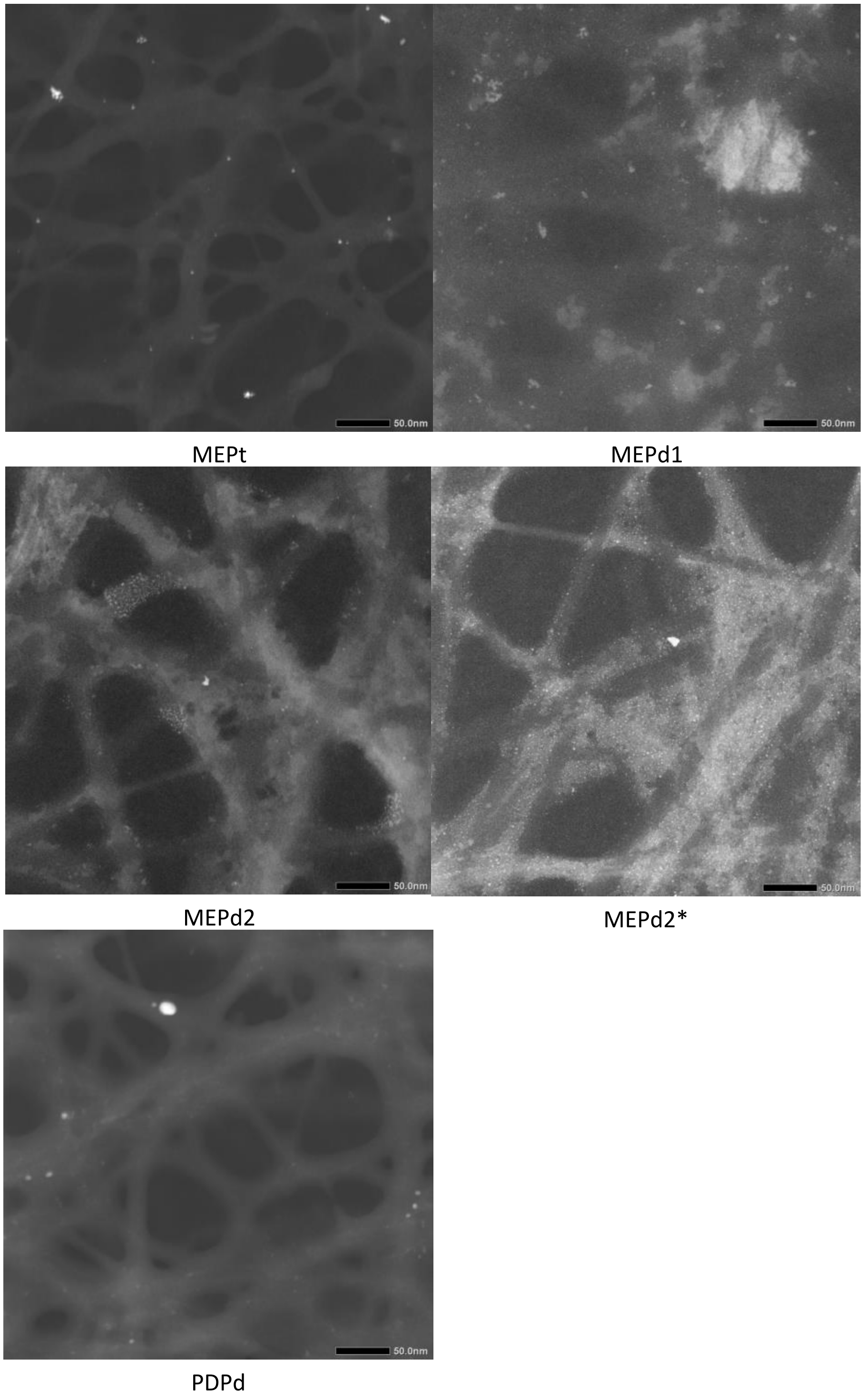

Figure S1. TEM images of MEPt, MEPd1, MEPd2, MEPd2* (after a hydrogenation experiment), and PDPd (scale bar 50 nm).

2. EDX sprectra

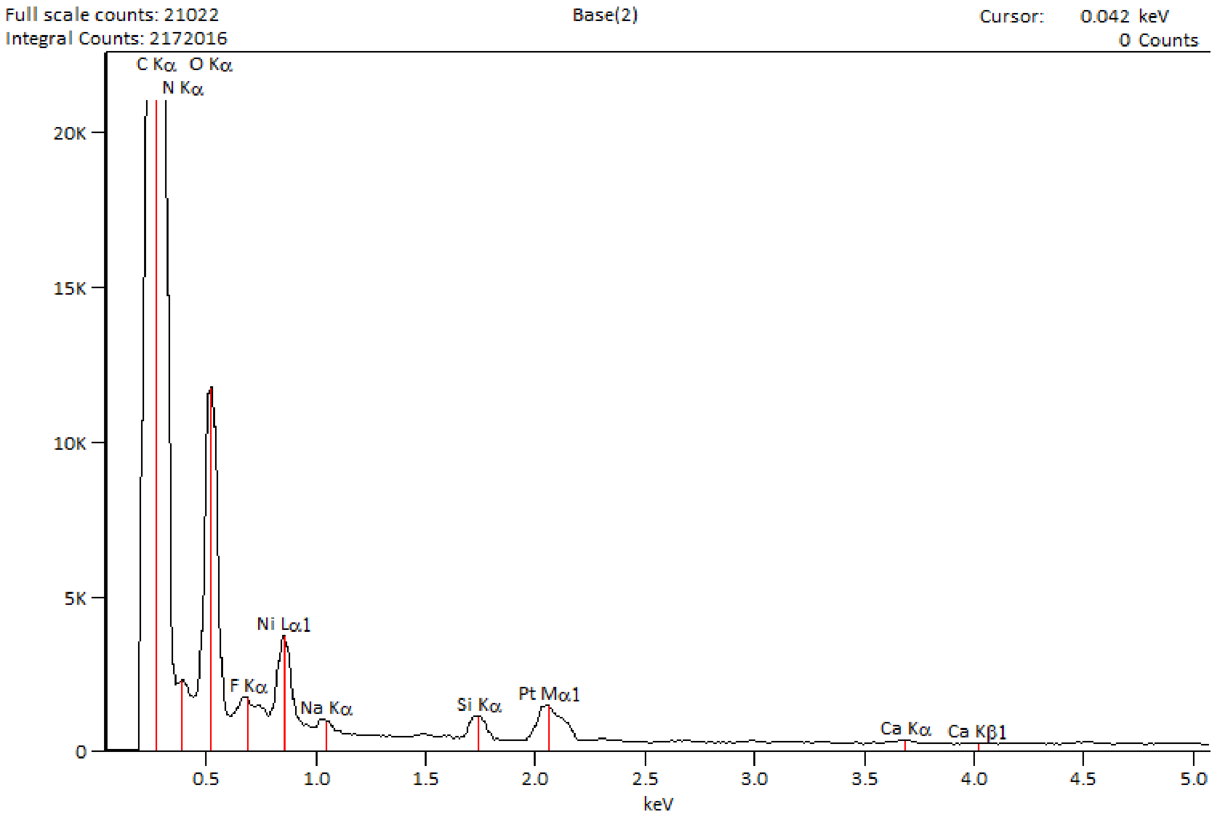

Figure S2. Full EDX spectrum of PDPt.

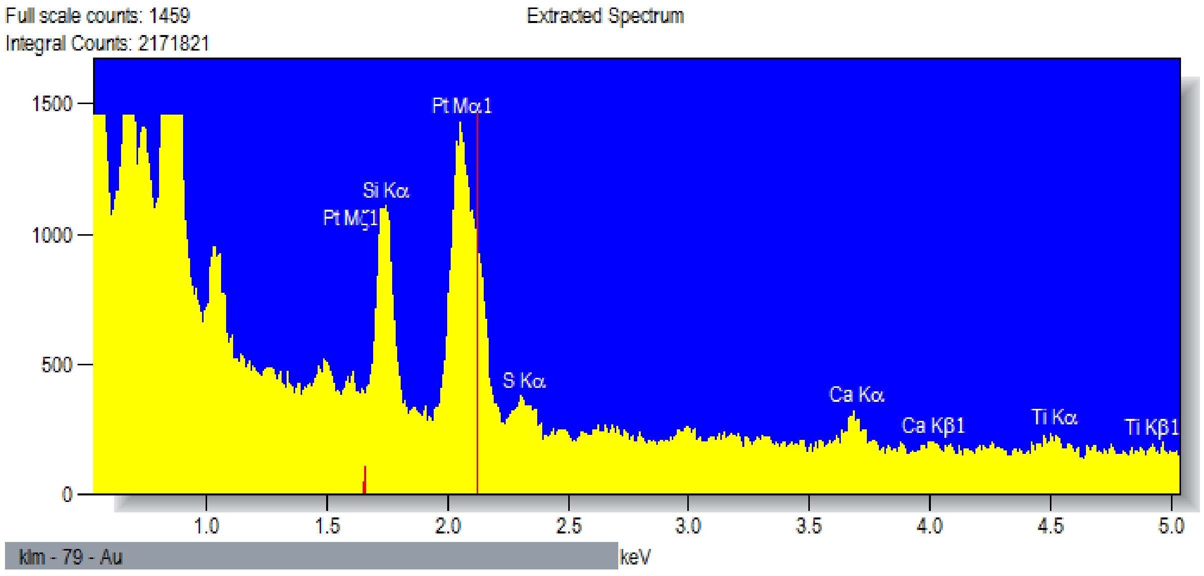

Figure S3. Excerpt of the EDX spectrum of PDPt.

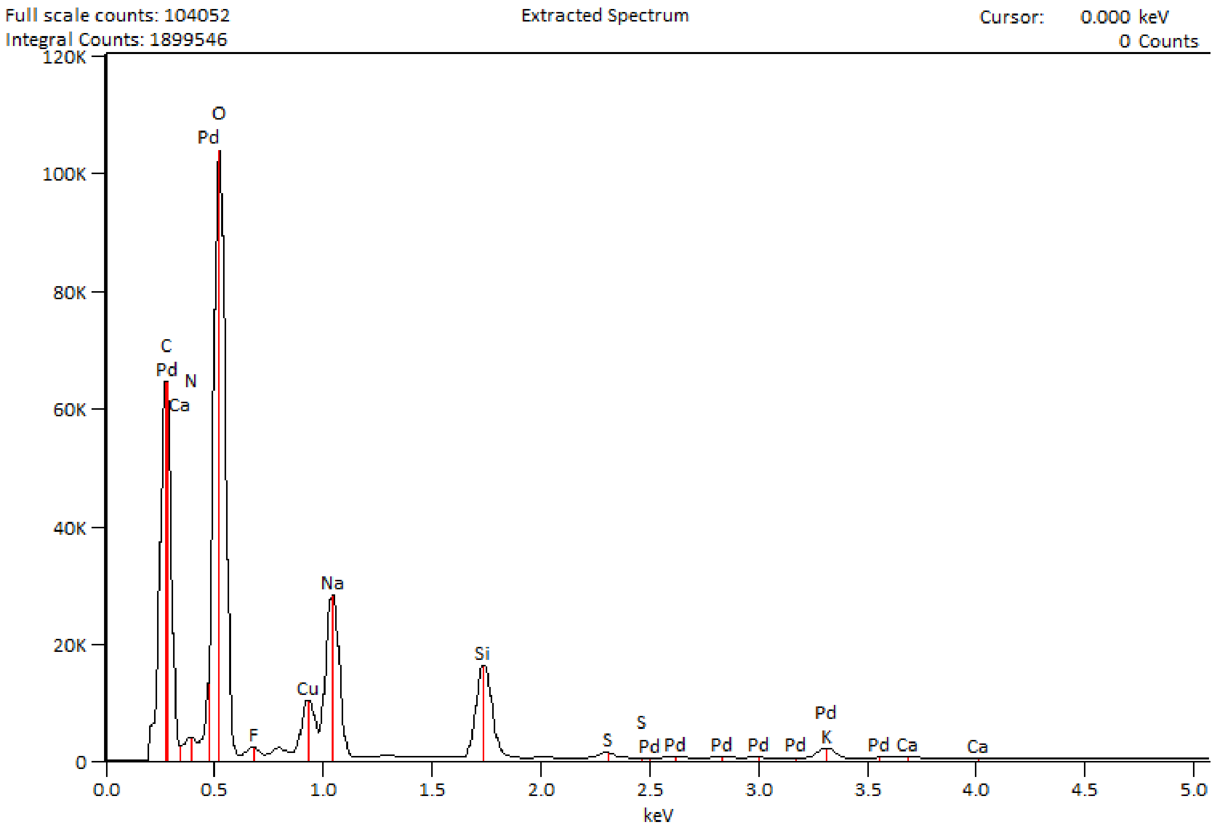

Figure S4. Full EDX spectrum of PDPd.

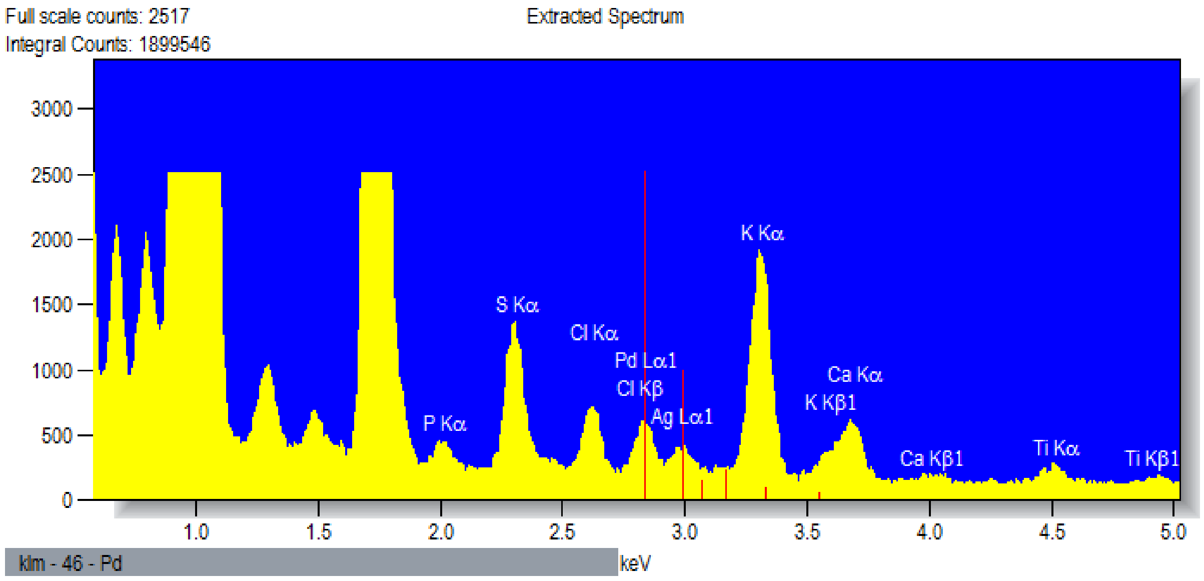

Figure S5. Excerpt of the EDX spectrum of PDPd.

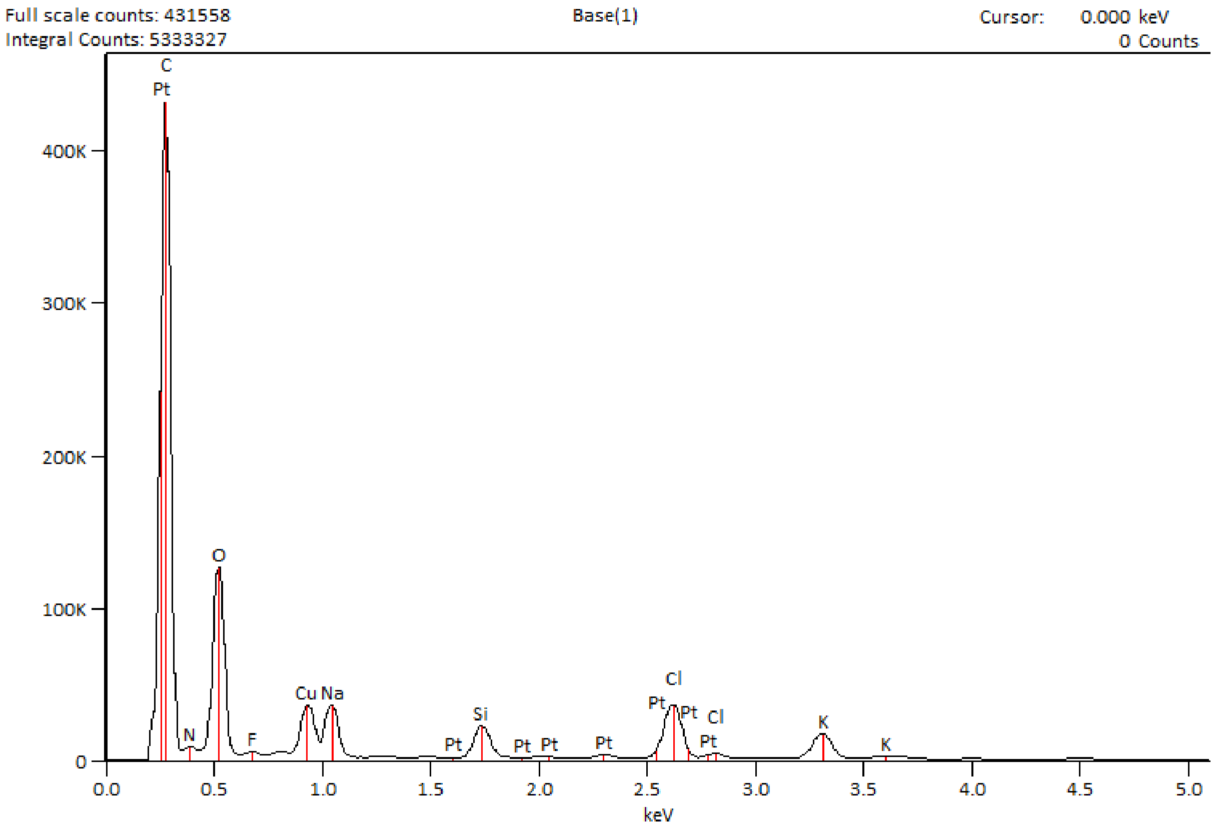

Figure S6. Full EDX spectrum of MEPt.

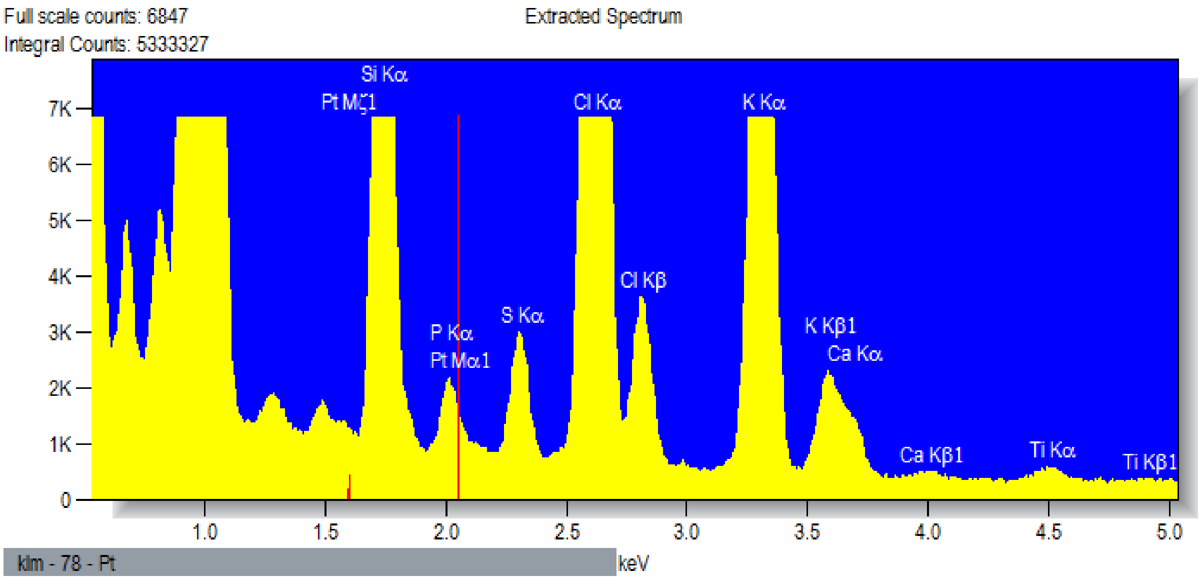

Figure S7. Excerpt of the EDX spectrum of MEPt.

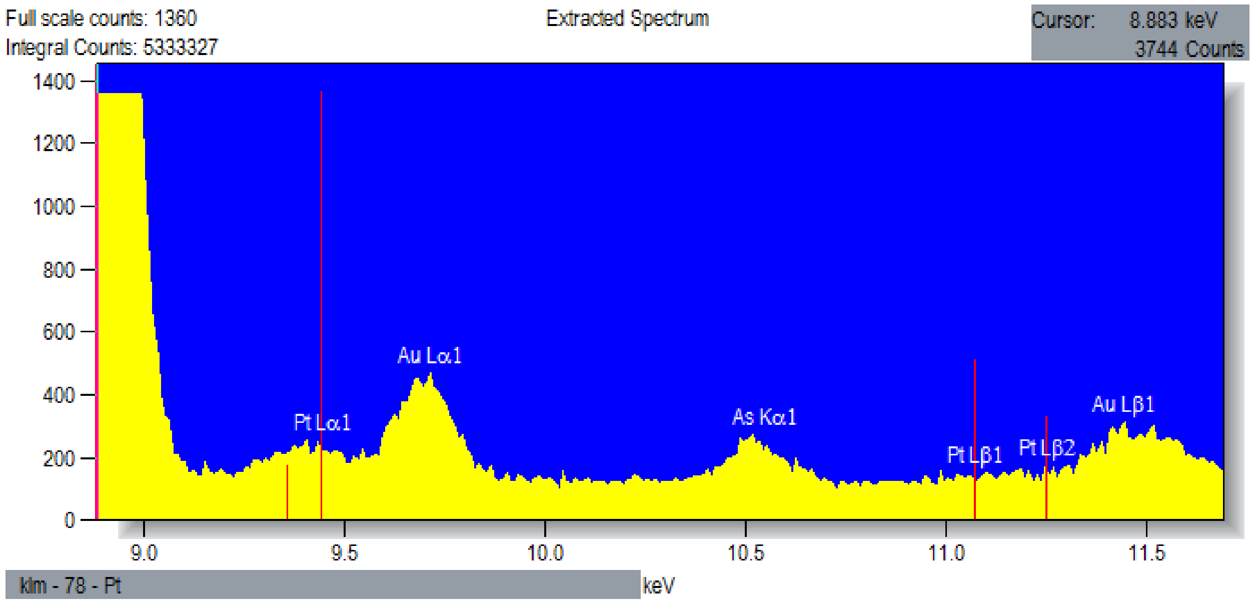

Figure S8. Excerpt of the EDX spectrum of MEPt.

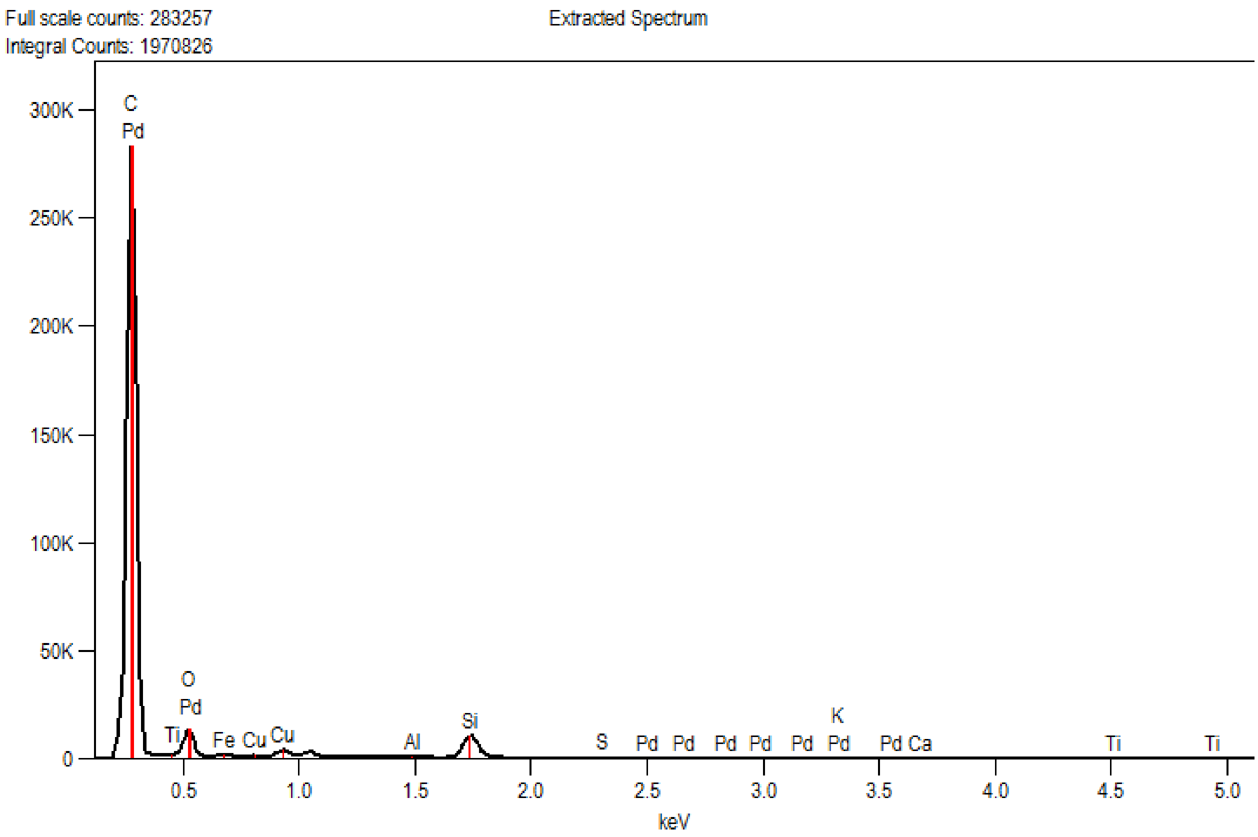

Figure S9. Full EDX spectrum of MEPd1.

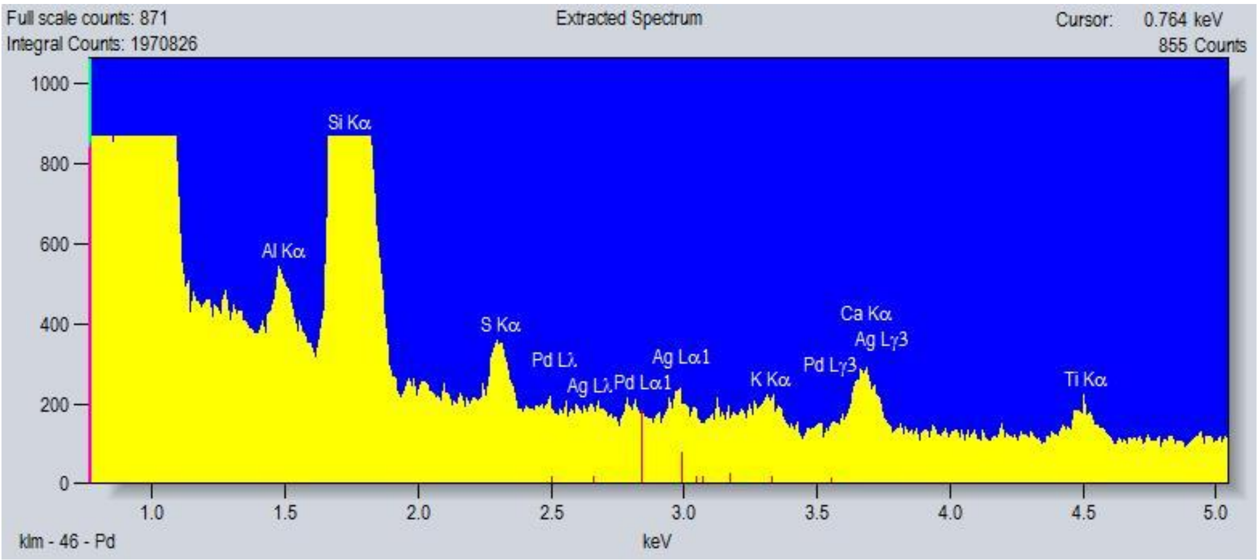

Figure S10. Excerpt of the EDX spectrum of MEPd1.

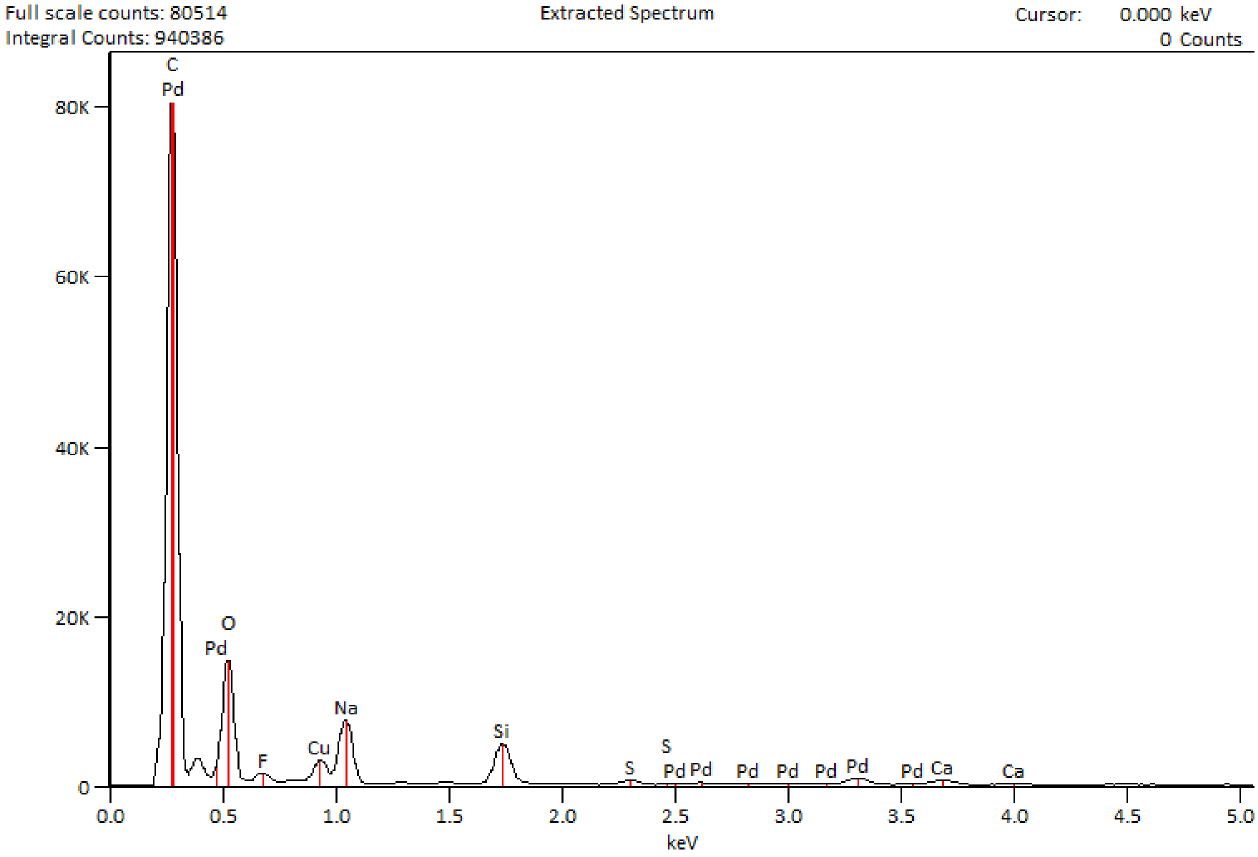

Figure S11. Full EDX spectrum of MEPd2.

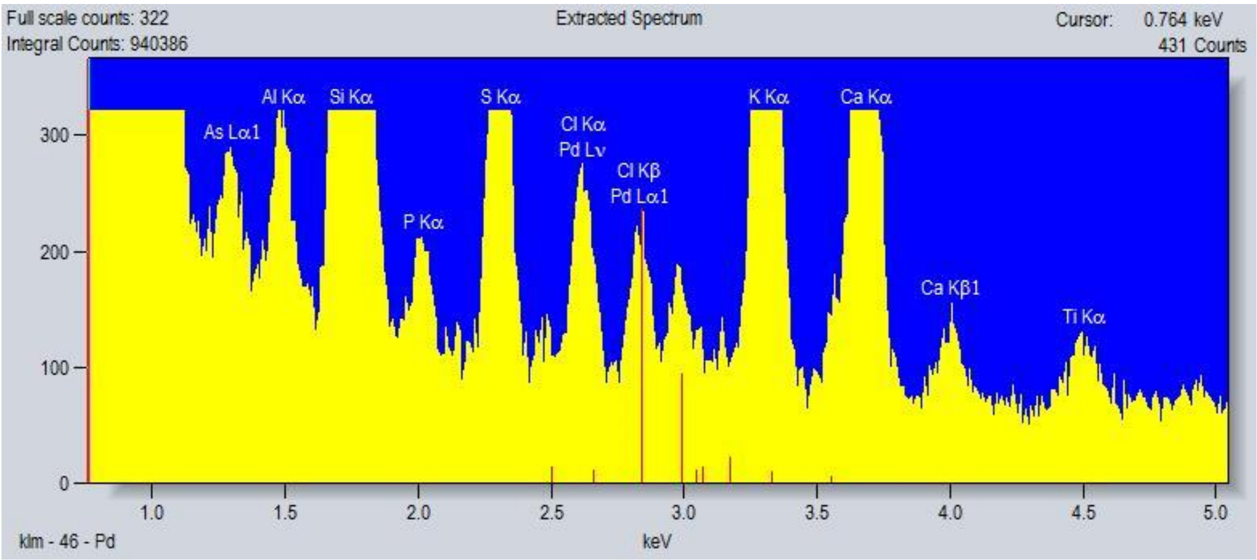

Figure S12. Excerpt of the EDX spectrum of MEPd2.

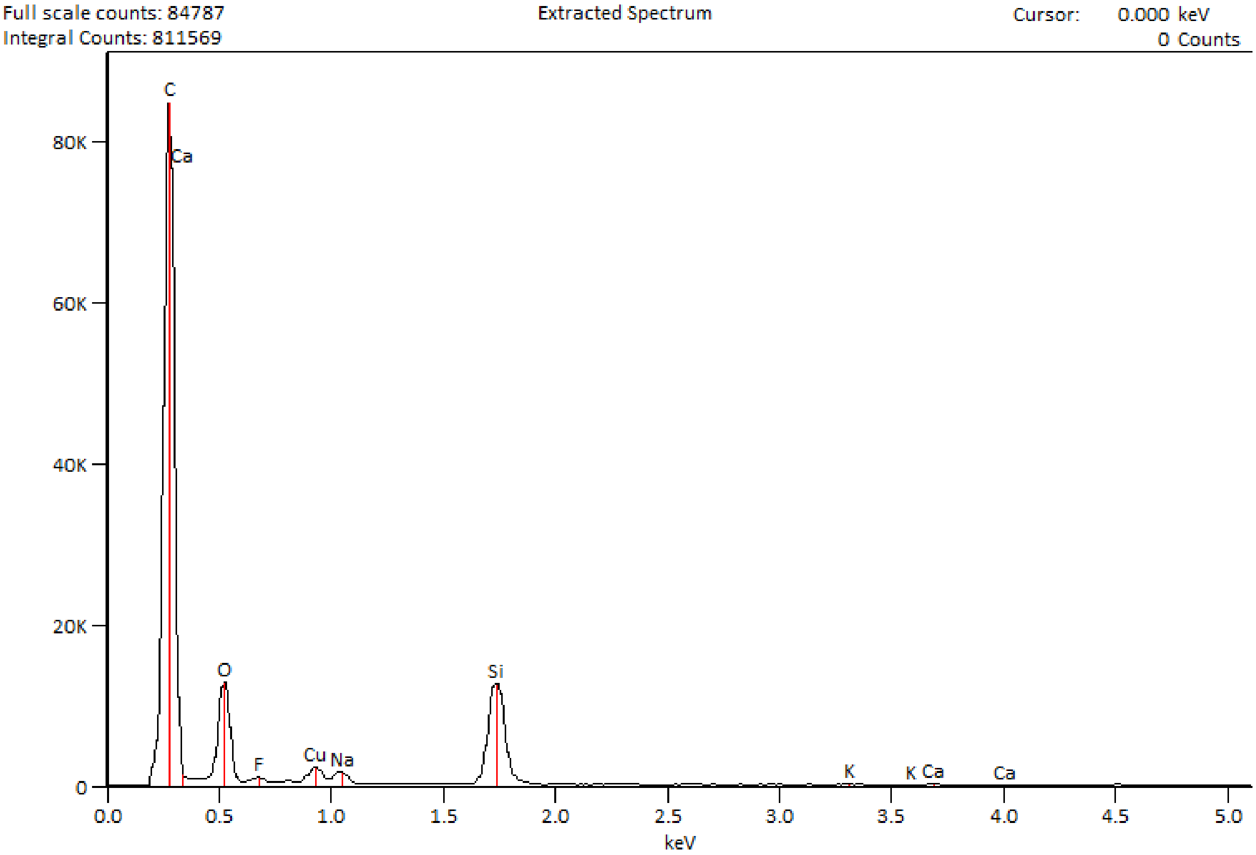

Figure S13. Full EDX spectrum of MEPd2 after hydrogenation.

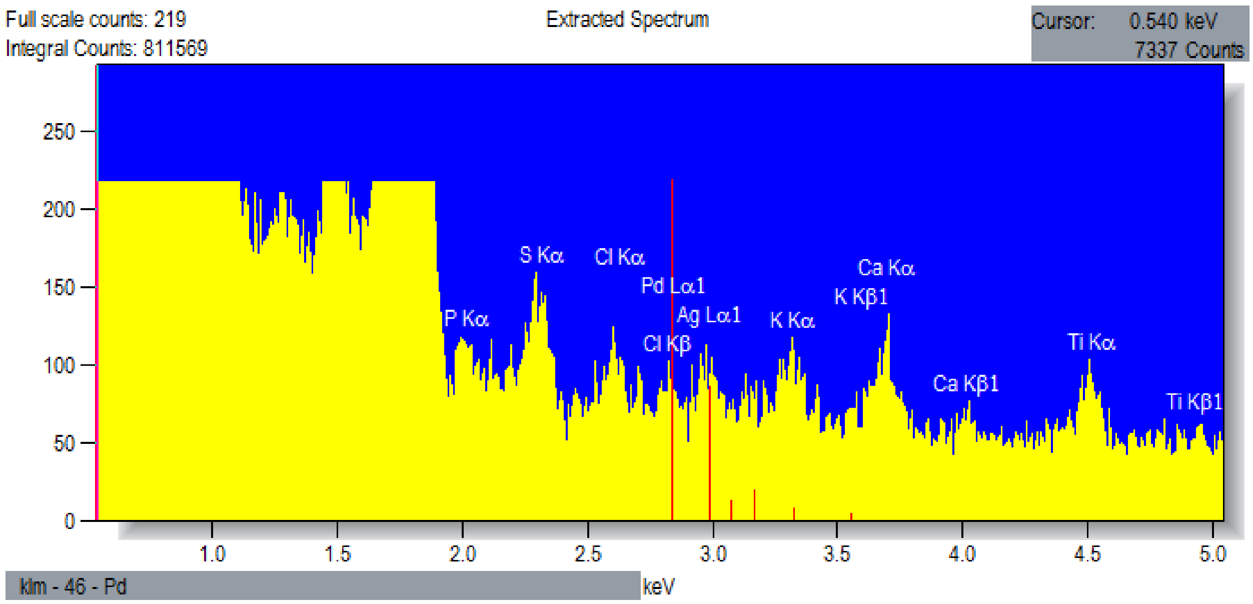

Figure S14. Excerpt of the EDX spectrum of Spectrum MEPd2 after hydrogenation.

3. Evaluation of hydrogenation experiments

The hydrogenation of ALB is a reaction network as shown in **Figure S15**. ALB can be directly hydrogenated to PB or first isomerized to cis-methyl styrene (CMS) or trans-methyl styrene (TMS), which are hydrogenated to PB. It was observed that both isomerization intermediates are produced in parallel and that PB is the only product of the hydrogenation, as also reported in the literature.[35] Under the selected reaction conditions, the hydrogenation of the aromatic ring is not possible.

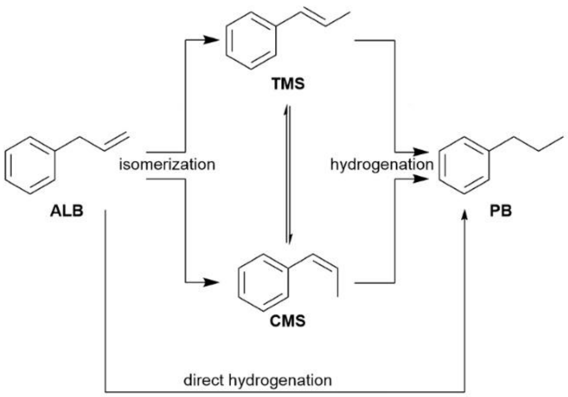

Figure S15. Mechanism of ALB hydrogenation to PB via isomerization to TMS and CMS or direct hydrogenation.

The hydrogen consumption at constant reactor pressure of about 1.1 bar was recorded for each ALB hydrogenation experiment. When hydrogen is consumed, ALB is converted to PB and ALB conversion can be calculated according to Equation (1).

$$\mathbf{X_E} = \frac{\mathbf{V_{H_2}(t)}}{\mathbf{V_{H_2}(t = \infty)}} \cdot \mathbf{X_{GC}} \qquad (1)$$

In Equation (1), $V_{H2}(t)$ is the consumed hydrogen volume at time t, $\mathbf{V_{H_2}(t = \infty)}$ is the total consumed hydrogen volume at the end of the reaction, and X_{GC} is the total conversion determined by gaschromatography (GC).

With the measured volume-time curve, the conversion-time curve can be obtained (**Figure S16**), from which the initial reaction rate r_0 (in mmol L^{-1} min^{-1}) was calculated according to Equation (2).

$$\mathbf{r_0} = \mathbf{c_{A0}} \cdot \left(\frac{\mathbf{dX}}{\mathbf{dt}}\right)_{\mathbf{X=10\%}} \qquad (2)$$

In Equation (2), c_{A0} is the initial concentration and dX/dt is the slope determined from the conversion profile. To calculate r_0, the conversion was fixed to 10%.

The catalyst activity A (mmol s^{-1} g^{-1}) was calculated from r_0 with Equation (3).

$$\mathbf{A} = \frac{\mathbf{r_0} \cdot \mathbf{V}}{\mathbf{m_{metal}}} \qquad (3)$$

In Equation (3), V is the reaction volume (0.1 L) and m_{metal} is the mass of used nanoparticles (active catalyst).

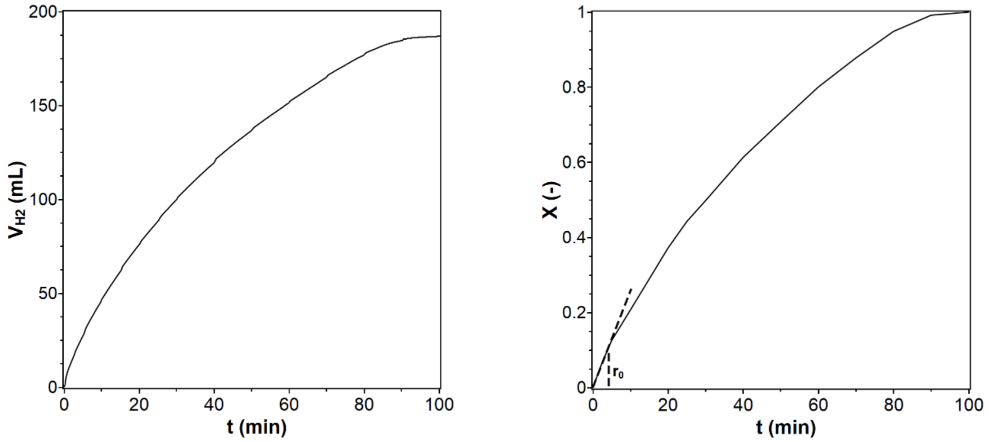

Figure S16. Cumulative hydrogen consumption (left) and respective conversion profile with the marked slope for the calculation of r_0 (right).

For further comparison, the turn-over-number (TON) and turn-over-frequency (TOF, h^{-1}) were calculated according Equation (4) and Equation (5), respectively.

$$\mathbf{TON} = \frac{\mathrm{n_{PB}}}{n_{Me}} = \frac{X \cdot \frac{m_{ALB}}{M_{ALB}}}{n_{Me}} \qquad (4)$$

$$\mathbf{TOF} = \frac{TON}{\mathrm{t_C}} \qquad (5)$$

In Equation (4), n_{PB} are the moles of produced propylbenzene and n_{Me} are the moles of used metal (Me = Pt, Pd). The molecular masses of Pt and Pd are 195.08 g mol^{-1} and 106.42 g mol^{-1}, respectively. 1 g ALB was used in most of the experiments. The molecular mass of ALB is 118.18 g mol^{-1}.

In Equation (5), t_c is a characteristic time. For comparing different catalysts, t_C was equal to t at X=10%, $t_{1/2}$ and 1h. This allows to compare the performance at three different points

within the reaction progress. For the recycling experiments, t_c was the time for achieving the maximum conversion in the first run.

4. ALB conversion profiles for investigated hydrogenation reactions

The ALB conversion profiles, which are used as base to calculate the reaction rates and to discuss the performance of the as-prepared cellulose-supported platinum and palladium are given below. The performance data are discussed in the main manuscript.

4.1 Variation of catalyst concentration, ALB concentration, and stirrer speed

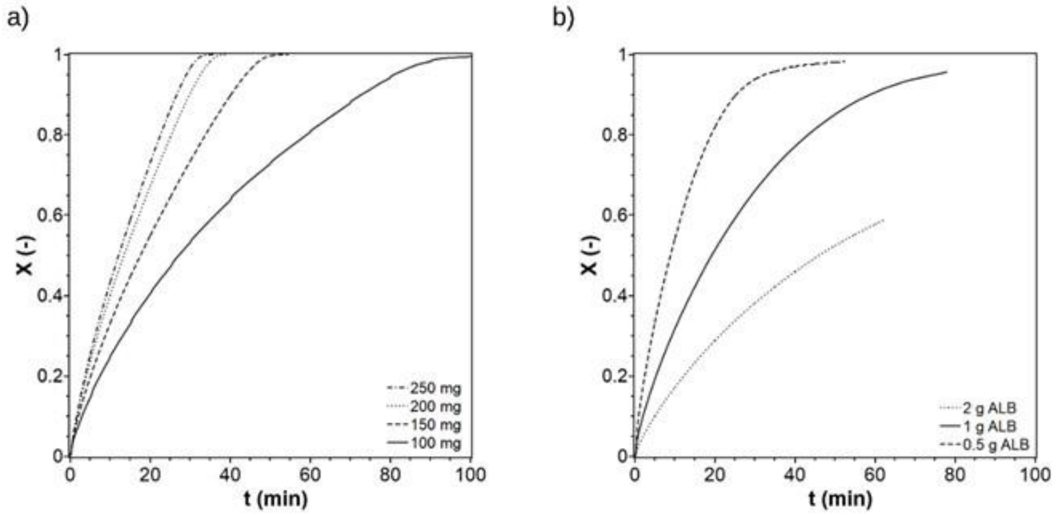

Figure S17. Conversion-time profiles using different MEPd1 amounts (a, 1 g ALB) and ALB concentrations (b, 100 mg MEPt).

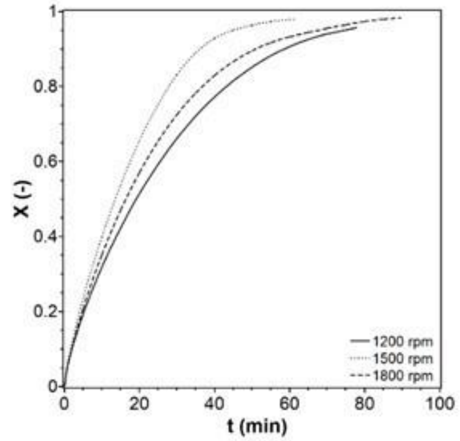

Figure S18. Concentration profiles for hydrogenation experiments with different stirrer speeds (100 mg MEPt, V = 100 mL, T = 25 °C, p = 1.1 bar).

4.2 Catalyst testing

ALB hydrogenation was carried out with different cellulose-supported Pd and Pt catalysts. The concentration profiles are shown in **Figure S19**. 100 mg of catalyst (except for 17 mg for PDPt and once 250 mg for MEPd1) and 1 g of ALB were used. The commercially available catalysts Pt@Al_2O_3 and Pd@Al_2O_3 were used as references.

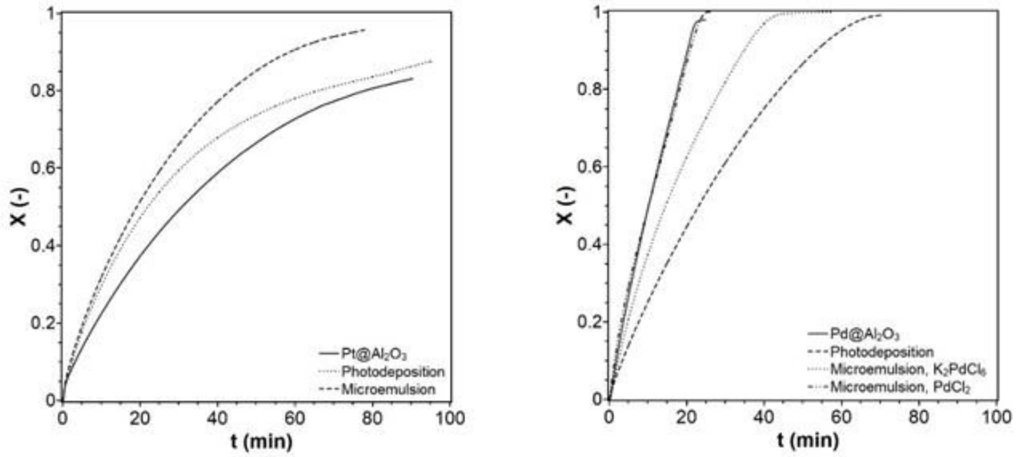

Figure S19. Conversion profiles of photodeposited and microemulsion deposited Pt@ModCe (PDPt, left) and Pd@ModCe (MEPd, right) with Pt@Al_2O_3 and Pd@Al_2O_3 as reference (n = 1200 rpm, V = 100 mL, T = 25°C, p = 1.1 bar, m_{ALB} = 1 g).

4.3 Catalyst recycling

PdPt, MEPd2, and Pd@Al_2O_3 have been exemplarily investigated for catalyst recycling. The conversion profiles for four consecutive runs are shown in **Figure S20**.

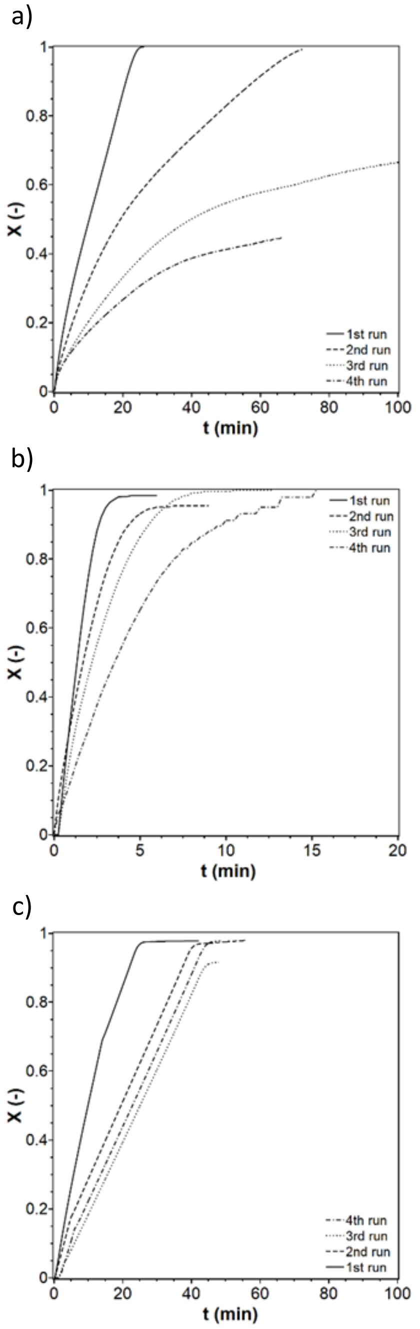

Figure S20. Conversion profiles for the recycling of MEPd2 (a, 100 mg, 1 g ALB), PDPt (b, 100 mg, 110 mg ALB), and Pd@Al_2O_3 (c, 100mg, 1 g ALB). PDPt, and Pd@Al_2O_3 were reloaded with ALB, and MEPd2 was washed and dried before reuse (n = 1200 rpm, V = 100 mL, T = 25°C, p = 1.1 bar).
